# Soil Charcoal to Assess the Impacts of Past Human Disturbances on Tropical Forests

**DOI:** 10.1371/journal.pone.0108121

**Published:** 2014-11-12

**Authors:** Jason Vleminckx, Julie Morin-Rivat, Achille B. Biwolé, Kasso Daïnou, Jean-François Gillet, Jean-Louis Doucet, Thomas Drouet, Olivier J. Hardy

**Affiliations:** 1 Service d'Évolution Biologique et Écologie, Faculté des Sciences, Université Libre de Bruxelles, Brussels, Belgium; 2 Laboratoire de Foresterie des Régions tropicales et subtropicales, Unité de Gestion des Ressources forestières et des Milieux naturels, Gembloux Agro-Bio Tech, Université de Liège, Gembloux, Belgium; 3 Laboratory of Wood Biology & Xylarium, Royal Museum for Central Africa, Tervuren, Belgium; 4 Laboratory of Applied Ecology, University of Abomey-Calavi, Cotonou, Benin; 5 Laboratoire d'Écologie Végétale et Biogéochimie, Faculté des Sciences, Université Libre de Bruxelles, Brussels, Belgium; Cirad, France

## Abstract

The canopy of many central African forests is dominated by light-demanding tree species that do not regenerate well under themselves. The prevalence of these species might result from ancient slash-and-burn agricultural activities that created large openings, while a decline of these activities since the colonial period could explain their deficit of regeneration. To verify this hypothesis, we compared soil charcoal abundance, used as a proxy for past slash-and-burn agriculture, and tree species composition assessed on 208 rainforest 0.2 ha plots located in three areas from Southern Cameroon. Species were classified in regeneration guilds (pioneer, non-pioneer light-demanding, shade-bearer) and characterized by their wood-specific gravity, assumed to reflect light requirement. We tested the correlation between soil charcoal abundance and: (i) the relative abundance of each guild, (ii) each species and family abundance and (iii) mean wood-specific gravity. Charcoal was found in 83% of the plots, indicating frequent past forest fires. Radiocarbon dating revealed two periods of fires: “recent” charcoal were on average 300 years old (up to 860 BP, n = 16) and occurred in the uppermost 20 cm soil layer, while “ancient” charcoal were on average 1900 years old (range: 1500 to 2800 BP, n = 43, excluding one sample dated 9400 BP), and found in all soil layers. While we expected a positive correlation between the relative abundance of light-demanding species and charcoal abundance in the upper soil layer, overall there was no evidence that the current heterogeneity in tree species composition can be explained by charcoal abundance in any soil layer. The absence of signal supporting our hypothesis might result from (i) a relatively uniform impact of past slash-and-burn activities, (ii) pedoturbation processes bringing ancient charcoal to the upper soil layer, blurring the signal of centuries-old Human disturbances, or (iii) the prevalence of other environmental factors on species composition.

## Introduction

For a long time, many tropical forests have been viewed as “virgin” or “primary” ecosystems, undisturbed by anthropogenic activities. However, increasing evidence from different continents has suggested that these forests may actually hide influences of past Human disturbances [1]. In South-East Asia for example, it has been suspected that agricultural activities developed as early as 7000 BP in Papua New Guinea [2] and 8000 BP in Thailand [3], in areas covered today by lowland uninhabited rainforests. In Northwest Belize, Ross [4] demonstrated that modern tree species composition appeared to display important differences between areas of high and low settlement of ancient Maya, because of different intensities of past forest gardening (recruitment of useful species). Similarly, in central Amazonia, “terra preta” soils covering an area of about 500 km^2^, on which stands apparent “pristine” rainforests, have resulted from intense burning and agricultural activities occurring about 2500 BP, which have considerably enhanced the fertility of these soils and may thus have impacted floristic diversity [5].

In central African moist forests, accumulating evidences show that Human has had a profound impact on the vegetation dynamics during the three last millennia. While only sparse evidence is recorded for Human presence in central Africa during the early and middle Holocene [6], [7], archaeological surveys have suggested a dramatic expansion of an ancestral Bantu population coming from the southern part of the actual Cameroon-Nigeria border during the third millennium BP [8]. Archaebotanical data have indicated that this expansion coincided with an increasing seasonality in the precipitation regime that would have generated a disruption of the forest cover and replaced it by savannas or open forest formations [9], [10], and therefore facilitated the Human colonization of central Africa [11], [12]. Direct evidence from pollen and diatom frequency diagrams have demonstrated increasing aridity in the region [13], [14] during the second half of the third millennium BP (especially between 2400 and 2100 BP), while charred botanical remains from this period have indicated that Bantus introduced the culture of pearl millet (*Pennisetum glaucum*, Poaceae), which requires a prolonged dry season [15]. Moreover, charcoal dating and identification have revealed higher abundance of pioneer species characterizing open forest formations [16]. The dryer areas of Central Africa were then more subject to fires during the dry season, but even when more humid conditions returned, the forest did not regenerate immediately as fires continued to maintain an open vegetation. According to archeological data, these resilient fires may not have been naturally caused only but also prompted by slash-and burn agricultural activities introduced by Bantu farmers [16], [17]. For some unclear reasons, a dramatic decline of Human occupation in Western Central Africa occurred about 1400 BP [7], [18], and it is only around the late middle age (about 600 BP) that evidence for Human activities are found again in the region [6], [19].

Nowadays, while natural openings are scarce in Central African moist forests, the upper canopy is often dominated by long-lived light-demanding tree species which seem to suffer from low regeneration rate underneath their own shade [20]. This has been explained by a decrease of forest clearing activities since Human settlements from deep forests zones have been forced to move along roadsides during the colonial period [21]. Considering these facts and the massive amounts of evidence (potsherds, charcoals, iron objects) recorded in the literature for ancient Human occupation in vast uninhabited regions of Central African forest [7], [15], it has been suggested that until a recent past, the widespread practice of slash-and-burn agriculture by farmers may have favored the competitiveness of light-demanding species and profoundly influenced the dynamics of tree diversity organization.

The pioneer works of Jones [22], [23] in the mahogany forest of Okomu (Nigeria) were among the firsts to link vegetation and past Human disturbances in African rainforests. Jones suggested that the abundance of old light-demanding species he observed resulted from intense past Human activities [19], [22]. Mahogany forests found in Cameroon [24], Central African Republic [25], [26], Republic of Congo [26] and Democratic Republic of the Congo [27] have been suspected to hide a similar history to those of Okomu [28]. More recently, Biwolé et al. [29] have emphasized coincidence between populations of light-demanding trees species and the age of the last Human disturbances in a forest of Southern Cameroon.

Therefore, if past slash-and-burn agricultural practices favored the establishment of light-demanding species and if their intensity was spatially heterogeneous, we expect a correlation between forest stand composition and signs of past Human disturbances, such as soil charcoal abundance. To our knowledge, this expectation has never been tested using an adequate statistical sampling design in tropical forests. To bridge this gap, we aimed to test the hypothesis that Human disturbance intensity varied in time and space and favored the establishment of long-lived light-demanding tree species in Central Africa. To do so, we have carried out tree inventories and soil charcoal analyses at a landscape scale in three rainforest areas located in Southern Cameroon, using soil charcoal abundance as a proxy for past slash-and-burn activities. We addressed the following specific questions: (1) Is charcoal abundance spatially structured throughout the landscape and according to soil depth? (2) What is the age distribution of charcoal and is age related to soil depth? (3) What is the spatial structure of floristic and functional properties of local tree assemblages, considering: (i) species abundance, (ii) relative abundance of three regeneration guilds (pioneer, non-pioneer light-demanding, shade-bearer), and (iii) wood-specific gravity (i.e. g.cm^−3^ of wood, used as a proxy for shade-tolerance)? (4) Does soil charcoal abundance explain variation in local tree assembly properties?

## Materials and Methods

### Study area

**Figure 1:**
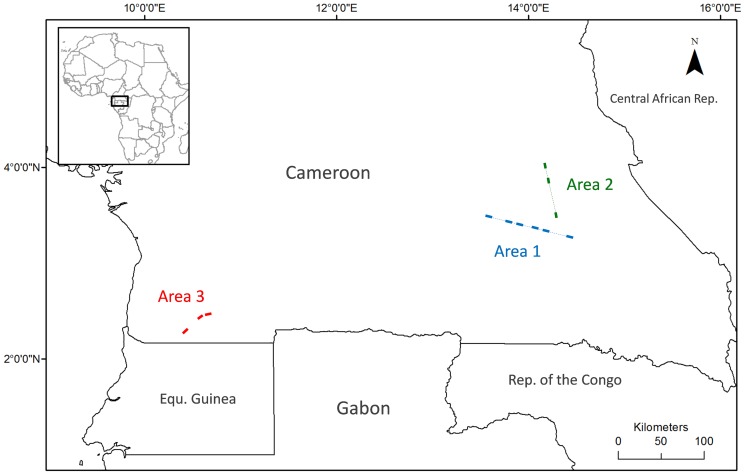
Geographical location of the three study areas. Each site is represented by a rectangle. Sites in Areas 1 and 2 are linearly disposed along a virtual transect represented by a dashed line.

Study areas corresponded to three forest logging concessions. Two of them, hereafter named Area 1 and Area 2, were located in South-East Cameroon while another, Area 3, was located in the South-Western part of the country (Figure 1). The vegetation of these areas consisted of transition formations between lowland evergreen and semi-evergreen moist tropical forest [24], where the upper canopy was dominated by typical long-lived light-demanding species like *Pericopsis elata* (Fabaceae) in Area 1, *Triplochiton scelroxylon* (Malvaceae) in Area 2 and *Lophira alata* (Ochnaceae) in Area 3. Topography was characterized by hilly and highly weathered plateaus displaying dense hydrographic networks, situated at an elevation of ca. 700 m a.s.l. in Areas 1 and 2, and ca. 500 m a.s.l. in Area 3. The climate within all study areas is equatorial, with mean annual temperatures of ca. 24–25°C and mean annual rainfall reaching ca. 1600–1700 mm (www.climatedata.eu). Rainfall displays a bimodal distribution, with a rainy season stretching from September to November and another from March to June, separated by two relatively less humid periods. Ferralsols constituted the most dominant type of soil, with substantial occurrence of Acrisols in Area 3 [30]. Permission to carry out our field inventories were provided by logging companies (contact details are given in Appendix S1).

### Inventory sites

A total of twelve sites were inventoried, of which six were located in Area 1, three in Area 2 and three in Area 3 (Figure 1). Each site corresponded to a linear transect along which 11 to 20 rectangular plots (40×50 m) were set up every 250 m and geo-referenced. This linear disposition of the plots within sites was designed to be able to apply torus-translation tests [31] (see details below). In Areas 1 and 2, sites were disposed along virtual transects extending over 101.5 and 66.5 km, respectively, while in Area 3, sites were irregularly disposed. Sites coordinates and the number of plots per site are given in Table S1 in File S1.

### Charcoal abundance

At each corner and at the centre of each plot, we excavated a soil pit with an auger (5 cm diameter), collecting soil samples at the following depth layers (in cm): 0–10, 10–20, 20–40, 40–60, 60–80 and 80–100. The two uppermost layers were smaller in order to analyze charcoal abundance at finer resolution, as we suspected that charcoal reflecting recent Human disturbance were mostly located in these superficial soil layers. Soil charcoal abundance is classically quantified by determining its mass after sieving the soil volume in water [32], [33]. This procedure being too time-consuming in the field for the large number of measures needed, we opted for a faster yet less precise method. Therefore, for each soil volume (280 cm^3^ for 20 cm of vertical profile), we searched manually for charcoals remains and visually attributed a “charcoal abundance index” (CAI) defined as follows: 0 =  absence of charcoal; 1 =  charcoal traces (scattered pieces or powder) and/or ≤5 pieces with a length <5 mm or 1 piece ≥5 mm; 2 =  more than 5 pieces with a length <5 mm and/or more than 1 piece ≥5 mm. CAI could not be estimated for layers deeper than 20 cm in site 4, due to logistic constraints. Within each of the three investigated areas, CAI was estimated by the same person (JV, JMR and ABB in Area 1, 2 and 3, respectively).

We validated our CAI method in two ways. First, we tested the correlation between our CAI and the real charcoal mass for 36 soil volumes (each of 140 cm^3^) excavated with an auger in a plot where previous digging revealed important charcoal presence (plot nr 2 of site 1, Area 1). For each volume, we first estimated the CAI, and then used a sieve of 2 mm mesh to collect all the charcoal pieces contributing significantly to the charcoal mass. We observed a significant correlation between CAI and ln(1+charcoal mass, in mg) (*r*-Pearson  = 0.91, *P*<0.001) and no overlap of charcoal mass distributions between CAI values (see Appendix S2). Second, as Chabal [34] showed that the total number of charcoal particles found in a soil volume is highly correlated to the total charcoal mass, we tested the correlation between our CAI and the number of charcoal particles counted for the same soil volume on (i) 1510 soil volumes sampled in Area 2 where charcoals collected by hand were systematically counted, and (ii) 900 soil samples collected for another project (in Area 1) for which charcoals were collected using a sieve of 2 mm mesh. Pearson correlations between CAI and ln(1+charcoal particles number) reached 0.92 and 0.93 in both datasets (P<0.001, Appendix S2).

### Radiocarbon dating

We selected 60 charcoal samples found at depth ranging from 5 to 150 cm collected in 37 random pits. In eight soil pits, charcoal samples from 2 to 5 different soil layers were dated to better assess the age-depth relationship. ^14^C dating was performed at the Poznań Radiocarbon Laboratory (Poland). Calibration was performed under the OxCal v4.1.7 program [35]–[37] with the IntCal09 atmospheric calibration curve [38] and expressed in BP.

### Species functional traits

Within each plot, we inventoried all the trees displaying a diameter at breast height (dbh) ≥20 cm. Over the three study areas, we found on average 32 individuals, 19 species, 18 genera and 13 families per plot. Table 1 summarizes abundance and diversity data for each study area. Species diversity was computed as the effective number of species expected in a random sample of *k* = 2 or *k* = 100 individuals [39], in order to give more weight to abundant and rare species, respectively. For all tree species identified in our plots, we compiled information on two key functional traits reflecting their shade-tolerance: regeneration guild (RG) and wood–specific gravity (WSG). Although well-defined classification of species in RG does not exist, three guilds could be defined, based on Hawthorne [40] and field observations in Central Africa (J. L. Doucet, A. Fayolle & J-F Gillet, pers. obs.; www.coforchange.eu): (i) pioneer species (P) require large gaps for establishment, (ii) non-pioneer light-demanding species (NPLD) can establish in shade but need a gap in the forest to grow to their full height, while (iii) shade-bearer species (SB) can be found in shade both as young and older plants. The relative abundance of each guild was computed for each of the 208 plots, after discarding unclassified species. WSG (g/cm^3^) was used because this variable reflects the diameter growth rate (see references in Slik [41]), as fast-growing (light-demanding) species are more likely to have lighter wood than slow-growing (shade-bearer) species [41], [42]. The WSG of most species could be assessed using a database [43]. When WSG data was missing for a species we assigned it the average WSG of its congeneric species, based on previous evidence showing that WSG is phylogenetically conserved [44]. For each plot, we calculated the mean WSG weighted by species relative abundances. The number of species for which the RG and WSG was known, as well as the abundance of each RG within each study area is represented in Table 1. A complete list of species, their corresponding RG and WSG, and their abundances in each study area is given in Table S2 in File S1.

**Table 1:** Abundance and diversity data for each study area.

	Area 1	Area 2	Area 3
Nr of 0.2-ha plots	120 (24 ha)	53 (10.6 ha)	35 (7 ha)
Nr of stems	3799	1699	1348
Total nr of identified ind.	3531 (93%^a^)	1693 (99.5%)	1335 (99%)
Nr of species	186	183	147
Dominant species	GS (8.2%^a^)	GS (8.3%)	BW (5.5%)
Nr of families	46	45	42
Dominant family	Annonaceae (15.5%^a^)	Annonaceae (20.0%)	Fabaceae (11.9%)
ENS(2)	51.2	50.5	54.1
ENS(100)	79.1	78.5	71.7
Nr of ind. assigned to a RG	3442 (97.5%^a^)	1576 (93.1%)	1263 (93.7%)
Nr of sp. assigned to a RG	172 (92.0%^b^)	155 (84.7%)	135 (91.8%)
Nr of ind. with WSG	3222 (91.2%^a^)	1577 (93.1%)	1217 (90.3%)
Nr of sp. with WSG	170 (91.4%^b^)	162 (88.5%)	131 (89.1%)
Nr of P	707 (20.5%^c^)	469 (29.8%)	261 (20.7%)
Nr of NPLD	761 (22.1%^c^)	306 (19.4%)	261 (20.7%)
Nr of SB	1974 (57.4%^c^)	801 (50.8%)	741 (58.7%)

RG  =  regeneration guild. P  =  Pioneers. NPLD  =  Non-Pioneer Light-Demanders. SB  =  Shade-bearers. ENS(2) or ENS(100)  =  effective number of species expected for a random sample of 2 or 100 individuals. Ind.  =  individuals. Sp.  =  species. WSG  =  wood-specific gravity (g/cm^3^). GS  =  *Greenwayodendron suaveolens* (Annonaceae). BW  =  *Blighia welwitschii* (Sapindaceae).

a Percentage calculated over the total number of stems in the study area.

b Percentage calculated over the total number of species in the study area.

c Percentage calculated over the number of individuals assigned to a RG.

### Spatial patterns

For each study area, landscape differences between sites were tested using Kruskal-Wallis tests for the following variables calculated at the plot level: (i) mean CAI in two soil layers (see next section of materials and methods), (ii) relative abundance and relative basal area of each RG, (iii) mean WSG and (iv) the abundance of each sufficiently represented species and family (≥20 individuals). Tests were performed using function kruskal.test from R stats package [45]. Finer scale spatial patterns were assessed within each of the three areas by measuring the spatial autocorrelation of these variables using Moran's *I* statistic [46], here defined as:
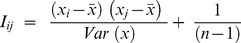
where *x_i_* and *x_j_* are the values of variable *x* for samples *i* and *j*, respectively; 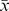 and *Var*(*x*) are, respectively, the mean and variance of variable *x*; *n* is the total sample size. The second term is a sample bias correction, ensuring that the average *I_ij_* over all existing *i*, *j* pairs is equal to zero. Autocorrelograms, representing the mean *I_ij_* values for a set of spatial distance intervals against distance, allowed a visual characterization of spatial patterns. Mantel tests [47] between the *I_ij_* matrix and the spatial distance matrix were used to assess the significance of spatial structure for each variable.

We analyzed the spatial autocorrelation of charcoal abundance using the CAI index obtained for each soil sample to assess patterns at contrasted spatial scales: within a soil pit (distinguishing adjacent, i.e. in contact, and non-adjacent soil layers), within a plot, within a site and among sites. At the plot level, spatial autocorrelation was also assessed for each RG relative frequency, mean WSG and the abundance of each sufficiently represented species and family. All spatial autocorrelation analyses were performed using software Torocor (Torocor 1.0, http://ebe.ulb.ac.be/ebe/Software.html) (see Appendices S3 and S4).

### Testing correlation between light-demanding species and charcoal abundance

**Figure 2:**
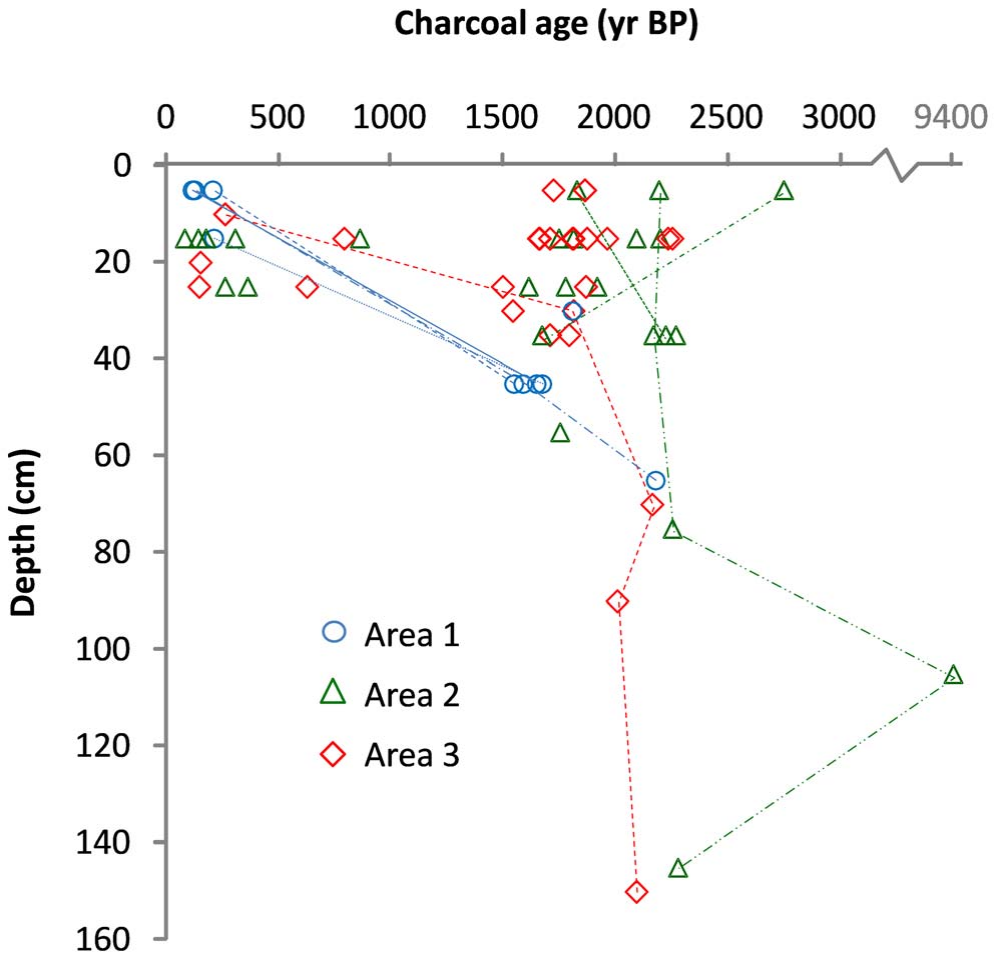
Age-depth relationship for 60 charcoal fragments sampled in the three areas investigated. Charcoals collected in a same soil pit are connected by a dashed line.

^14^C dating revealed a bimodal distribution in charcoal age: 16 out the 60 (27%) samples were dated between 80 and 860 BP (hereafter named “recent” charcoals, relatively speaking) and were only found in the upper soil layer (up to 25 cm), while 44 of them (73%) were dated more than 1500 BP (“ancient” charcoal) and found in the whole soil profile (Figure 2). Therefore, we estimated the abundance of soil charcoals at the plot level by computing, respectively, (i) the mean CAI value over both the 0–10 and 10–20 cm soil layers (proxy for “recent” fires) and (ii) the mean CAI value over the soil layers deeper than 20 cm (proxy for “ancient” fires).

For each study area taken individually, we then tested the correlation (at the plot level) between the mean CAI above and below 20 cm depth and: (1) the relative frequencies and relative basal areas of each RG, (2) the abundance of each species and family represented by at least 20 individuals (in the study area), and (3) the mean WSG. As classical correlation test is likely to be too liberal when analyzing spatial data, we tested the correlations using (i) a classical parametric test and (ii) a non-parametric torus-translation procedure taking spatial autocorrelation into account [31]. The latter consisted of generating randomized datasets where the correlation between variables was broken down while the spatial autocorrelation of each variable was kept intact. More specifically, within each site independently, the mean CAI values of the 20 plots were permuted among plots while keeping the original spatial order of plots (except at the extremities). This was like considering that the 20 plots of a site were situated along a ring and that CAI values were shifted by a random number of steps along the circle. 4999 of such permutations, independently repeated for each of the sites of a given area, were conducted to obtain null distributions of Pearson's correlation coefficients (computed using all plots). This allowed defining 95% confidence envelopes under the null hypothesis that there was no correlation between local CAI values and other variables. A test was significant when the observed correlation coefficient (i.e. without permutation) lied outside the 95% confidence envelope. By conserving the local structure (within site) of variables when permuting the objects, the torus-translation procedure tested the correlation at finer scale (i.e. within 3 to 5 km long transects) than the classic correlation test. Torus-translation analyses were performed using software Torocor (see Appendix S4).

## Results

### Charcoal ages and distribution

Charcoal was found as powder or entire fragments from 1 to 10 mm. It occurred at higher abundance between 20 and 60 cm in Area 1, between 10 and 40 cm in Area 2, and between 10 and 60 cm in Area 3 (see Appendix S3). It was present in 72, 83 and 94% of the plots between 0 and 20 cm deep in Areas 1, 2 and 3, respectively (89, 92 and 94% between 20 and 100 cm).

Radiocarbon dating revealed two distinct periods of ancient fires (Figure 2): 16 charcoal pieces were dated from 80 to 860 BP (hereafter “recent” charcoal), while 43 pieces were dated from 1495 to 2745 BP (hereafter “ancient” charcoal), and one sample was dated 9400 BP. All recent charcoal samples were found in the upper 25 cm soil layer, while ancient charcoal was found at all depths. Beside this trend between recent and ancient charcoal, there was no relationship between age and depth for ancient charcoals, even within a same soil pith (Figure 2). Calibration details for all dated samples are available in Table S3 in File S1.

**Figure 3:**
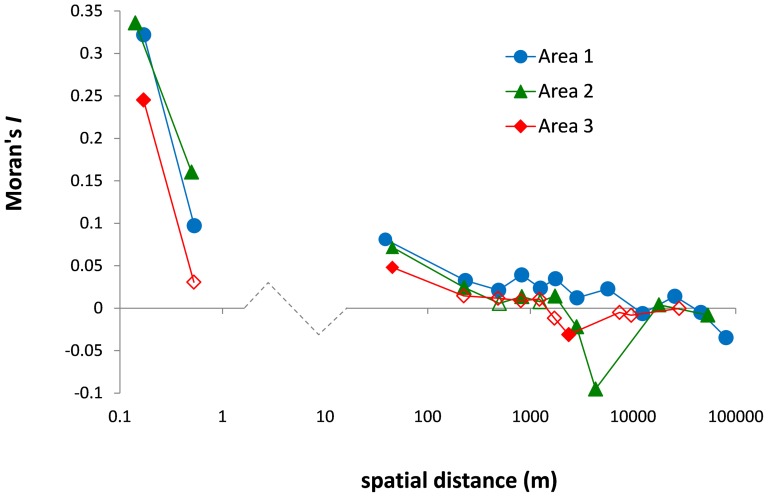
Autocorrelogram of CAI values for each study area: mean Moran's *I* computed for 12 to 14 distance intervals. On the left side (<1 m), the abscissa for the two first symbols represent the vertical distance between soil layers from a same pit, the left and right symbols distinguishing adjacent layers from non-adjacent layers, respectively. On the right side (>10 m), the abscissa corresponds to horizontal distance between soil volumes from different pits located in a same plot (first symbol between 10 m and 100 m), different plots of a same site (between 100 m and 5000 m) or different sites (>5000 m). Full symbols indicate significantly positive or negative Moran's *I* value (*P*<0.05).

For each study area, CAI values within soil pits were much more similar between adjacent soil layers than between non-adjacent ones (Figure 3, left). The correlation between mean plot CAI in soil layers above and below 20 cm depth was highly significant in Areas 1 and 2 (*r*-Pearson  = 0.56 and 0.51, respectively; *P*<0.001 under bilateral torus-translation test) but not in Area 3 (*r*-Pearson  = −0.09; *P* = 0.66). Among soil pits, there was a regular decrease of Moran's *I* values with the logarithm of the spatial distance within each study area (Figure 3, right). In particular, Moran's *I* values were always significantly positive between pits from a same plot, demonstrating a heterogeneity in charcoal abundance among plots within each area, although the relatively weak values found (<0.1) also indicate a high heterogeneity of charcoal abundance at a very local scale (i.e. within plot). At larger spatial scales, considering plot level mean CAI values separately in the 0–20 cm and 20–100 cm soil layers, Mantel tests between Moran's *I* and spatial distance matrices were highly significant for both soil layers in Area 1 (*P*<0.01; see Appendix S5) while no spatial structure was found in Areas 2 and 3. Consistently, these mean CAI values differed significantly among sites only in Area 1, where charcoal was more abundant in the western part (sites 1 to 3) than in the eastern part (sites 4 to 6; *P*<0.001, see Table 2).

**Table 2:** Mean charcoal abundance index (CAI) and functional trait variables per site, and Kruskal-Wallis tests for among sites differences within each study area (using values computed at the plot level).

	CAI^a^		Functional traits						
	0–20	20–100	WSG^b^	P-a	NPLD-a	SB-a	P-b	NPLD-b	SB-b
Site 1	0.29	0.37	0.62	0.29	0.28	0.43	0.34	0.36	0.31
Site 2	0.29	0.44	0.64	0.18	0.32	0.50	0.24	0.43	0.35
Site 3	0.16	0.27	0.66	0.15	0.20	0.65	0.19	0.35	0.45
Site 4	0.18	.	0.62	0.25	0.27	0.47	0.31	0.37	0.31
Site 5	0.11	0.30	0.65	0.16	0.16	0.68	0.28	0.21	0.51
Site 6	0.14	0.16	0.62	0.24	0.14	0.62	0.41	0.20	0.41
***p*** **-value** c	***	***	***	**	***	***	**	***	**
Site 7	0.47	0.35	0.59	0.32	0.20	0.48	0.47	0.23	0.30
Site 8	0.35	0.28	0.58	0.35	0.22	0.44	0.43	0.26	0.31
Site 9	0.56	0.41	0.64	0.24	0.15	0.61	0.37	0.22	0.41
***p*** **-value**	0.43	0.39	***	*	0.16	***	0.31	0.77	*
Site 10	0.40	0.38	0.62	0.19	0.27	0.55	0.26	0.42	0.32
Site 11	0.42	0.36	0.65	0.23	0.15	0.62	0.28	0.25	0.47
Site 12	0.38	0.55	0.64	0.22	0.20	0.58	0.27	0.32	0.41
***p*** **-value**	0.78	0.19	0.39	0.53	**	0.46	0.97	*	0.18

Sites 1 to 6, 7 to 9, and 10 to 12 correspond to Areas 1, 2 and 3, respectively.

a Charcoal abundance index (0–20 cm and 20–100 cm).

b Wood-specific gravity (g/cm^3^).

c *P*-value of a Kruskal-Wallis test among sites: *p-value <0.05, **p-value <0.01, *** *p*-value <0.001. P/NPLD/SB  =  Pioneers/Non-Pioneer Light-Demanders/Shade-Bearers. a  =  relative abundance. b  =  relative basal area.

### Floristic and functional traits spatial patterns

In Area 1, differences between sites and spatial autocorrelations were significant (*P*<0.05) for each RG relative abundance and relative basal area, and for the mean WSG per plot, while fewer significant differences (Table 2) and spatial structures (Appendix S5) were observed within Areas 2 and 3. More specifically, the relative abundance and basal area of NPLD and P were not spatially structured in Area 2, whereas in Area 3, significant spatial autocorrelation was only observed for NPLD relative abundance and relative basal area. The proportion of species and families displaying significant spatial structure ranged between 35% and 65% within each study area (see Appendix S5). For each area, Kruskal-Wallis tests for difference of species abundances and family abundances between sites are given in Table S4 in File S1.

### Correlations between vegetation characteristics and charcoal abundance

In Area 1, the abundance of charcoal in both the 0–20 cm and 20–100 cm soil layers (dominated by “recent” and “old” charcoals, respectively) and the relative abundance and basal area of NPLD decreased from West to East (sites 1 to 6), while the relative abundance and basal area of SB displayed the reverse pattern (Figure S1). Accordingly, Pearson correlations between NPLD relative abundance/relative basal area and CAI in each soil layer (measured at the plot level) were statistically significant using classical correlation tests (Table 3). However, these correlation coefficients became non-significant when using torus-translation tests which tested for within-site correlation and corrected for spatial autocorrelation. In Areas 2 and 3, all tests of correlation between vegetation characteristics and charcoal abundance were non-significant (Table 3). Finally, no significant correlation was observed between CAI and the abundance of any species or family in any study area, regardless of the test considered (see Appendix S6).

**Table 3:** Pearson correlations between CAI in two soil layers (0–20 cm and 20–100 cm) and variables related to species functional traits.

	Area 1	Area 2	Area 3
	r^b^	r	r
**P**	−0.05	−0.03	0.08
	*−0.15*	*−0.21*	*0.00*
**NPLD**	0.20*	−0.26	0.24
	0.28***	*0.10*	*−0.13*
**SB**	−0.09	0.19	−0.26
	*−0.07*	*0.15*	*0.11*
**P relative basal area**	−0.18	−0.07	−0.04
	*−0.20*	*−0.12*	*−0.06*
**NPLD relative basal area**	0.19	−0.08	0.24
	0.28**	*0.06*	*−0.14*
**SB relative basal area**	0.00	0.17	−0.21
	*−0.06*	*0.08*	*0.20*
**WSG** a	0.02	−0.01	0.16
	*−0.10*	*0.12*	*0.08*

P/NPLD/SB  =  relative abundance of Pioneers/Non-Pioneer Light-Demanders/Shade-Bearers.

a Wood-specific gravity (g/cm^3^).

b *r*-Pearson correlation between row variable and CAI in the (i) 0–20 cm (upper line) and (ii) the 20–100 cm (bottom line; values in italics) soil layers. “*”indicates significant test with the classic correlation test: *p< = 0.05 **p<0.01 ***p<0.001. All the tests based on toroidal translations were non-significant.

## Discussion

### Origin of soil charcoal: a bimodal age distribution

Charcoal was found in the majority of the plots in each study area and in every soil layers, although at a higher frequency for the layers lying between 10 or 20 cm and 60 cm deep, which is consistent with previous studies [48]. Radiocarbon dating revealed two periods of ancient fires, with 16 samples dated between 80 to 860 BP (“recent” charcoals), and 43 samples between 1500 to 2200 BP (plus one dated 9400 BP; “ancient” charcoals). This bimodal age distribution has already been observed in previous ^14^C dates distributions from other Central African countries [16], [48]. It is also consistent with previous archeological evidence from Gabon and Cameroon pointing out high Human population density during the third millennium BP up to a few centuries afterwards, followed by a gap of Human occupation during the Middle Age [6], [19]. This congruence between the abundances of charcoal and archeological artifacts through time supports the hypothesis that charcoal would mainly result from human-mediated fires. Ancient charcoals were found throughout the whole soil profile while recent ones were only found in the superficial soil layers (0–25 cm; Figure 2). For ancient charcoal, the absence of any age-depth relationship might be due to soil profile mixing (pedoturbation) resulting from biotic or abiotic agents. The main biotic agents in tropical soils are termites and ants [49]–[52] but earthworms [53], rodents, wind-fallen trees or tree uprooting can also play a role [54], while the main abiotic factor is colluvium [55] when located downhill or on a steep slope.

### Charcoal abundance displays significant spatial structure

Charcoal was found in all sites, suggesting that Human impacted the whole forest cover in every study areas, especially in Area 3, where charcoal was present in 94% of the plots. Its abundance, however, displayed significant spatial autocorrelation at different scales, reflecting differential intensity of past disturbance across each region. At the smallest spatial scale, charcoal abundance was highly correlated between adjacent layers of a same pit, but the correlation dropped sharply when non-adjacent layers were compared. Hence, charcoal tended to occur as relatively small aggregates in the soil. There was also a regular decay of spatial autocorrelation with the logarithm of the horizontal distance, indicating spatial heterogeneity among plots and sites. In Area 1, a substantial part of the spatial autocorrelation can be explained by an East-West increase in charcoal abundance among sites (Figure S1), which could reflect a landscape gradient (∼100 km) in the intensity of past slash-and-burn activities along the transect. In this area, as well as in Area 2, the correlation between charcoal abundance (at the plot level) in the 0–20 cm and the 20–100 cm soil layers was highly significant, according to both classic correlation tests and torus-translation tests (the latter testing within site correlation), indicating that the similarity of charcoal abundance between both layers occurred at the landscape scale (among sites) but also locally (within a site). Unfortunately, it cannot be assessed whether this correlation indicates that slash-and-burn agriculture was concentrated in the same places over the last centuries and c. 2000 years ago because the upper soil layer contains a mixture of both recent and ancient charcoal.

### Local distribution of light-demanding species does not seem to be explained by charcoal abundance

In all study areas, most of the identified species could be categorized in a RG and characterized by a WSG. The few uncategorized species may either have a non-well known ecology or very large ecological amplitudes for light conditions. At the plot level, the relative abundance or basal area of each RG, as well as the mean WSG, displayed significant spatial autocorrelation in Area 1, while only two to three of these variables were spatially structured in Areas 2 and 3 (see Appendix S5).

Under our initial hypothesis, slash-and-burn agriculture favored the establishment of pioneer (P) species, which were then replaced by non-pioneer light demanding (NPLD) species within less than a century and, eventually, shade-bearer (SB) species became dominant after a few centuries. Hence, we expected a positive correlation between CAI of the upper layer, reflecting partially the intensity of centuries-old fires, and the relative abundance of NPLD. In Area 1, the relative abundance of NPLD was indeed significantly positively correlated with the abundance of charcoals from the 0–20 cm (*r* = 0.2; *P* = 0.03) soil layer, but also with charcoal from the 20–100 cm (*r* = 0.28; *P* = 0.005) soil layer, when using a classic correlation test, not correcting for spatial autocorrelation. This is due to the parallel decay of charcoal and NPLD abundances among the six sites from West to East, which may have revealed a causal relationship if the intensity of past slash-and-burn agriculture had been stronger in the West. However, as such pattern has not been observed in the other two areas, and as toroidal translation tests also indicated that no correlation between RG relative abundance (and basal area) and soil charcoal abundance occurred within site, the among site correlation in Area 1 may well be spurious.

In Area 2 and Area 3, no significant correlations were detected. In fact, for the specific hypothesis that NPLD trees would have been favored by recent fires, we can note that while there is indeed a positive correlation between CAI in the upper soil layer and the relative abundance or basal area of NDPL in Area 1 and 3 (*r*-Pearson ranging from 0.19 to 0.24), the correlations become negative in Area 2 (*r*-Pearson ranging from −0.08 to −0.26). Hence, variation in floristic composition cannot be clearly explained by the abundance of soil charcoal.

We therefore suggest several explanations for the absence of significant signal. First, assuming a link between tree age and diameter for the entire community, we suspected that most of the trees of our dataset were too young to reflect the last Human disturbances, generating statistical noise. Nevertheless, correlation coefficients between light-demanding species frequencies and CAI did not increase after eliminating the smallest trees of our dataset (dbh <30, <40 <50, <60 and <70 cm, not shown).

Second, we assumed that charcoal from the superficial soil layer may sometimes be removed or covered by allochtonous materials due to colluvium processes, in such a way that it cannot be detected anymore. However, even after removing plots potentially subject to colluvium processes, i.e. located on a steep slope or downhill, correlation tests remained non-significant (not shown). Thus, if pedoturbation affects charcoal abundance, it is rather due to biotic agents (e.g. termites or ants). The latter could have “contaminated” the superficial soil layer (0–20 cm) with “ancient” charcoal, explaining why 59% of the dated charcoals found in the 0–25 cm soil layer were “ancient” (>1500 BP). By mixing with recent charcoals near the soil surface, ancient charcoals inevitably skewed our estimation of the abundance of “young” vs. “old” charcoals” (as recent and ancient material cannot be distinguished in the field) and therefore generated important statistical noise when testing our correlations.

Third, the absence of correlation may be due to an inappropriate study scale. Indeed, under our hypothesis, a correlation between charcoal abundance and the prevalence of light-demanding species is expected provided that there is sufficient spatial heterogeneity in the intensity of slash-and-burn agriculture to imprint a spatial heterogeneity in the abundance of light-demanding species. However, widespread and repeated slash-and-burn agriculture over the landscape may have favored light-demanding species on a large scale without causing substantial spatial heterogeneity on a more local scale.

Finally, it remains possible that the impact of past slash-and-burn agriculture on the floristic composition of forest stands is weak compared to the impact of other factors, like soil properties, climatic gradients (expected to be weak in our study areas), the history of forest establishment (e.g. forest regression ca. 3000 BP), or even stochastic processes structuring the distribution of species.

## Conclusions

Investigating the role of past Human disturbances on tropical tree communities had, to our knowledge, never been explored with an adequate statistical design based on soil charcoal. Charcoal remains were found in most of the plots in each study area, revealing frequent and widespread past forest fires in SE and SW Cameroon, presumably prompted by slash-and-burn agricultural activities. Fires occurred during two very distinct periods, one lying from ca. 80 to 850 BP and another from ca. 1500 to 2800 BP. The abundance of charcoal and non-pioneer light-demanding tree species were both spatially structured and positively correlated in only one of the three study areas. However, the reverse pattern was observed in Area 2 and correlations were non-significant at a local scale when correcting for spatial autocorrelation. Testing alternative hypotheses on the drivers of floristic composition should give more insights to confirm or not whether charcoal is an appropriate proxy to study the impacts of past Human disturbances, and whether these disturbances have significantly modified present-day tree species assemblages in Central African moist forests.

## Supporting Information

Figure S1 **Mean site values of each RG relative abundance and CAI in Area 1.** Values were computed at the plot level. Mean CAI was computed over two soil layers: 0–20 and 20–100 cm. Note that data are missing for the 20–100 cm soil layer in site 4. N-P  =  Non-Pioneer.
(TIF)

File S1 **Tables S1-S4.** Table S1 (in File S1) Sites Coordinates and number of plots per site. Table S2 (in File S1) Abundances and functional traits of all species inventoried in the three study areas. Table S3 (in File S1) Dates BP of 60 charcoal samples. Table S4 (in File S1) Mean sites values of each variable tested for their difference between sites, in each study Area.
(DOCX)

Appendix S1 **Contact details of logging companies.**
(DOCX)

Appendix S2 **Datasets used to calibrate the charcoal abundance index.**
(XLSX)

Appendix S3 **Files to be used in Torocor software for testing spatial autocorrelation of charcoal abundance at multiple spatial scales.**
(XLSX)

Appendix S4 **Plot values for spatial autocorrelation and correlation tests on Torocor.**
(XLSX)

Appendix S5 **Results of spatial autocorrelation tests for each variable of Appendix S4.**
(XLSX)

Appendix S6 **Pearson correlations between all variables, tested by a torus-translation procedure.**
(XLSX)
