# Supplementary figures and images for: Soil Charcoal to Assess the Impacts of Past Human Disturbances on Tropical Forests
Source: PLoS One. 2014 Nov 12;9(11):e108121. doi: 10.1371/journal.pone.0108121 (PMC4229094; doi:10.1371/journal.pone.0108121)

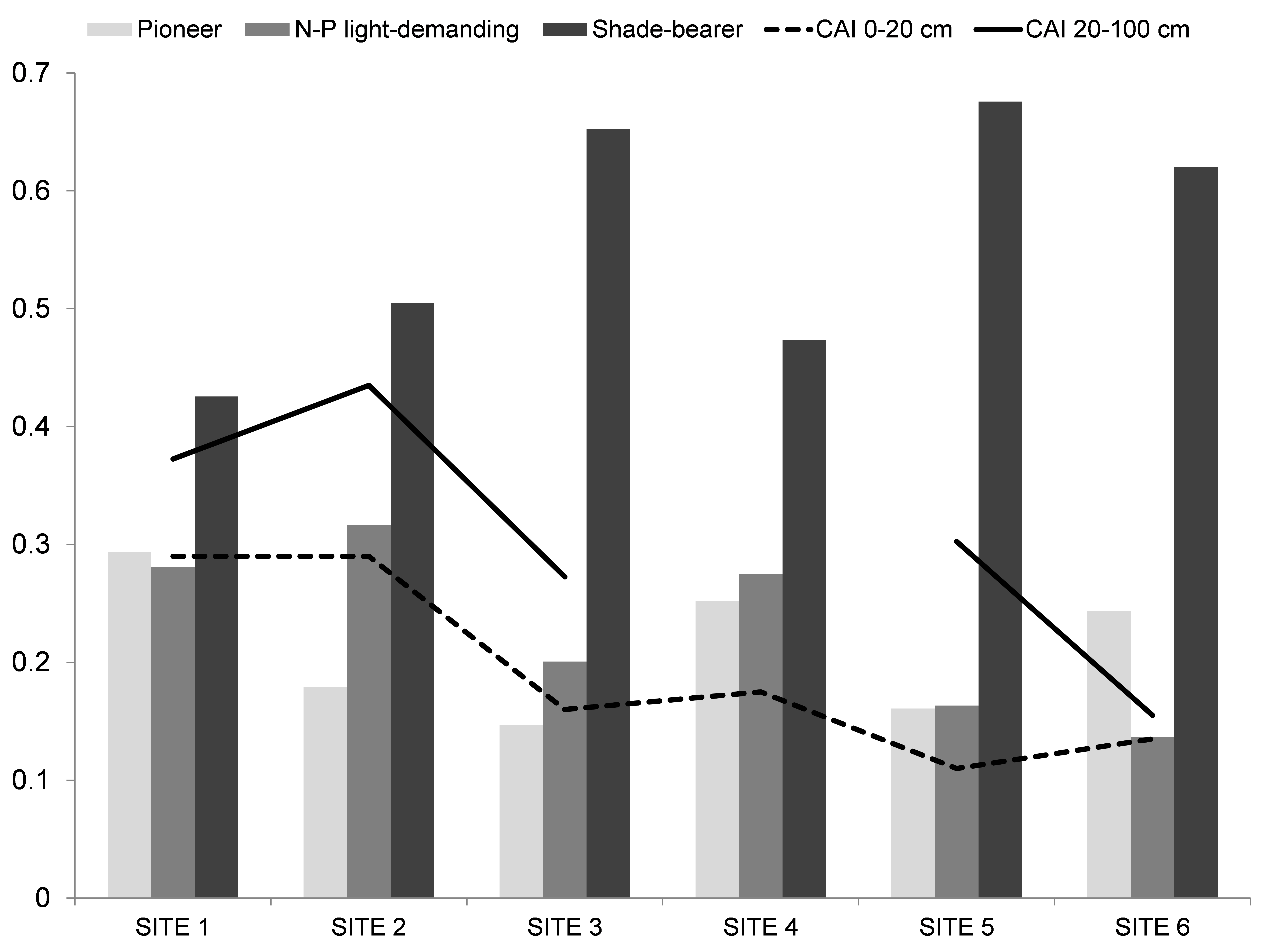

Supplement: Figure S1 — Mean site values of each RG relative abundance and CAI in Area 1. Values were computed at the plot level. Mean CAI was computed over two soil layers: 0–20 and 20–100 cm. Note that data are missing for the 20–100 cm soil layer in site 4. N-P = Non-Pioneer. (TIF) [file pone.0108121.s001.tif]
