# Supplementary material for: Soil Charcoal to Assess the Impacts of Past Human Disturbances on Tropical Forests
Source: PLoS One. 2014 Nov 12;9(11):e108121. doi: 10.1371/journal.pone.0108121 (PMC4229094; doi:10.1371/journal.pone.0108121)
Supplement: File S1 — Tables S1-S4. Table S1 (in File S1) Sites Coordinates and number of plots per site. Table S2 (in File S1) Abundances and functional traits of all species inventoried in the three study areas. Table S3 (in File S1) Dates BP of 60 charcoal samples. Table S4 (in File S1) Mean sites values of each variable tested for their difference between sites, in each study Area. (DOCX) [file pone.0108121.s002.docx]

**Supporting information (Tables)**

**Table S1.** Coordinates (dms) of site edges (in plot nr 1 and the last plot of the site) for each inventory site.

|  | **Area** | **Nr plots** | **Plot nr 1 edge** | **Last plot edge** |
| --- | --- | --- | --- | --- |
| **Site 1** | 1 | 20 | 3°29’0.4’’N 13°34’6.9’’E | 3°28’24.0’’N 13°36’44.8’’E |
| **Site 2** | 1 | 20 | 3°26’7.2’’N 13°46’39.6’’E | 3°25’30.1’’N 13°49’17.5’’E |
| **Site 3** | 1 | 20 | 3°24’14.0’’N 13°54’50.8’’E | 3°23’37.3’’N 13°57’28.0’’E |
| **Site 4** | 1 | 20 | 3°22’30.5’’N 14°2’18.2’’E | 3°21’54.1’’N 14°4’56.2’’E |
| **Site 5** | 1 | 20 | 3°20’46.1’’N 14°9’49.2’’E | 3°20’9.5’’N 14°12’26.5’’E |
| **Site 6** | 1 | 20 | 3°17’12.4’’N 14°25’14.1’’E | 3°16’35.7’’N 14°27’52.4’’E |
| **Site 7** | 2 | 19 | 4°02’05.4’’N 14°10’35.6’’E | 3°59’33.1’’N 14°11’03.3’’E |
| **Site 8** | 2 | 18 | 3°52’23.9’’N 14°12’59.9’’E | 3°49’58.4’’N 14°13’27.6’’E |
| **Site 9** | 2 | 16 | 3°29’07.0’’N 14°18’23.9’’E | 3°26’59.6’’N 14°18’52.2’’E |
| **Site 10** | 3 | 12 | 2°18’07.6’’N 10°26’08.8’’E | 2°17’09.5’’N 10°24’56.9’’E |
| **Site 11** | 3 | 11 | 2°26’04.9’’N  10°34’11.4’’E | 2°26’57.6’’N  10°35’29.3’’E |
| **Site 12** | 3 | 12 | 2°27’23.8’’N 10°38’55.8’’E | 2°28’00.9’’N 10°40’20.4’’E |

**Table S2.** Abundances and functional traits of all species inventoried in the three study areas.

| species | Family | RG^a^ | WSG^b^ | Area 1 | Area 2 | Area 3 | total |
| --- | --- | --- | --- | --- | --- | --- | --- |
| *Afrostyrax lepidophyllus* | Huaceae | SB | unknown | 88 | 4 | 0 | 92 |
| *Afzelia bipindensis* | Fabaceae | NPLD | 0.731 | 11 | 2 | 3 | 16 |
| *Afzelia pachyloba* | Fabaceae | NPLD | 0.683 | 0 | 0 | 1 | 1 |
| *Aidia micrantha* | Rubiaceae | SB | 0.663 | 3 | 0 | 0 | 3 |
| *Albizia adianthifolia* | Fabaceae | P | 0.508 | 43 | 4 | 0 | 47 |
| *Albizia ferruginea* | Fabaceae | P | 0.494 | 2 | 2 | 0 | 4 |
| *Albizia glaberrima* | Fabaceae | P | 0.567 | 0 | 12 | 1 | 13 |
| *Albizia sp.* | Fabaceae | P | 0.527 | 0 | 1 | 0 | 1 |
| *Albizia zygia* | Fabaceae | P | 0.510 | 0 | 2 | 0 | 2 |
| *Allanblackia floribunda* | Clusiaceae | SB | 0.687 | 8 | 1 | 1 | 10 |
| *Allanblackia kisonghi* | Clusiaceae | SB | 0.646 | 0 | 0 | 1 | 1 |
| *Allophylus sp.* | Sapindaceae | SB | 0.509 | 0 | 0 | 2 | 2 |
| *Alstonia boonei* | Apocynaceae | P | 0.321 | 41 | 55 | 22 | 118 |
| *Amphimas ferrugineus* | Fabaceae | NPLD | 0.673 | 3 | 0 | 0 | 3 |
| *Amphimas pterocarpoides* | Fabaceae | NPLD | 0.617 | 0 | 0 | 1 | 1 |
| *Angylocalyx pynaertii* | Fabaceae | SB | unknown | 78 | 4 | 0 | 82 |
| *Aningeria robusta* | Sapotaceae | NPLD | unknown | 0 | 1 | 0 | 1 |
| *Annickia affinis* | Annonaceae | SB | 0.437 | 65 | 50 | 63 | 178 |
| *Annona dimako* | Annonaceae | SB | 0.511 | 0 | 0 | 1 | 1 |
| *Annona sp.* | Annonaceae | SB | 0.511 | 0 | 0 | 12 | 12 |
| *Anonidium mannii* | Annonaceae | SB | 0.291 | 26 | 37 | 5 | 68 |
| *Anopyxis klaineana* | Rhizophoraceae | unknown | 0.800 | 14 | 2 | 1 | 17 |
| *Anthocleista schweinfurthii* | Gentianaceae | P | 0.523 | 2 | 0 | 0 | 2 |
| *Anthonotha fragrans* | Fabaceae | SB | 0.529 | 0 | 3 | 4 | 7 |
| *Anthonotha macrophylla* | Fabaceae | SB | 0.842 | 2 | 0 | 6 | 8 |
| *Antiaris welwitschii* | Moraceae | P | 0.389 | 0 | 0 | 1 | 1 |
| *Antidesma laciniatum* | Euphorbiaceae | SB | 0.650 | 0 | 7 | 1 | 8 |
| *Antidesma membranaceum* | Euphorbiaceae | SB | 0.650 | 0 | 5 | 0 | 5 |
| *Antrocaryon sp.* | Anacardiaceae | P | 0.502 | 4 | 0 | 0 | 4 |
| *Aoranthe cladantha* | Rubiaceae | P | 0.806 | 4 | 1 | 0 | 5 |
| *Araliopsis soyauxii* | Rutaceae | unknown | unknown | 0 | 0 | 1 | 1 |
| *Baillonella toxisperma* | Sapotaceae | NPLD | 0.725 | 3 | 0 | 0 | 3 |
| *Baphia sp.* | Fabaceae | SB | 0.701 | 2 | 0 | 0 | 2 |
| *Barteria fistulosa* | Passifloraceae | unknown | unknown | 0 | 2 | 3 | 5 |
| *Barteria nigritiana* | Passifloraceae | P | unknown | 1 | 0 | 0 | 1 |
| *Beilschmiedia sp.* | Lauraceae | SB | 0.574 | 20 | 9 | 4 | 33 |
| *Berlinia congolensis* | Fabaceae | SB | 0.617 | 0 | 0 | 21 | 21 |
| *Blighia sapida* | Sapindaceae | SB | 0.762 | 2 | 0 | 0 | 2 |
| *Blighia welwitschii* | Sapindaceae | SB | 0.786 | 4 | 6 | 74 | 84 |
| *Bobgunnia fistuloides* | Fabaceae | unknown | 0.866 | 1 | 2 | 0 | 3 |
| *Bombax buonopozense* | Malvaceae | P | 0.319 | 0 | 2 | 0 | 2 |
| *Brenania brieyi* | Rubiaceae | unknown | unknown | 0 | 4 | 0 | 4 |
| *Bridelia sp.* | Phyllanthaceae | P | 0.553 | 6 | 0 | 1 | 7 |
| *Calpocalyx dinklagei* | Fabaceae | NPLD | 0.723 | 18 | 1 | 26 | 45 |
| *Calpocalyx heitzii* | Fabaceae | NPLD | 0.727 | 0 | 0 | 1 | 1 |
| *Canarium schweinfurthii* | Burseraceae | P | 0.402 | 2 | 1 | 5 | 8 |
| *Carapa procera* | Meliaceae | SB | 0.604 | 0 | 0 | 11 | 11 |
| *Carapa sp.* | Meliaceae | SB | 0.560 | 19 | 4 | 0 | 23 |
| *Casearia* | Flacourtiaceae | NPLD | 0.508 | 0 | 0 | 1 | 1 |
| *Ceiba pentandra* | Malvaceae | P | 0.305 | 2 | 1 | 0 | 3 |
| *Celtis adolfi-friderici* | Cannabaceae | NPLD | 0.554 | 31 | 26 | 0 | 57 |
| *Celtis mildbraedii* | Cannabaceae | NPLD | 0.617 | 22 | 0 | 0 | 22 |
| *Celtis philippensis* | Cannabaceae | NPLD | 0.703 | 2 | 0 | 0 | 2 |
| *Celtis tessmannii* | Cannabaceae | NPLD | 0.656 | 19 | 3 | 4 | 26 |
| *Celtis zenkeri* | Cannabaceae | NPLD | 0.608 | 17 | 18 | 0 | 35 |
| *Centroplacus glaucinus* | Centroplacaceae | SB | unknown | 23 | 14 | 0 | 37 |
| *Chrysophyllum africanum* | Sapotaceae | NPLD | 0.646 | 0 | 5 | 0 | 5 |
| *Chrysophyllum boukokoense* | Sapotaceae | SB | 0.655 | 3 | 1 | 0 | 4 |
| *Chrysophyllum lacourtianum* | Sapotaceae | SB | 0.630 | 17 | 13 | 2 | 32 |
| *Chrysophyllum sp.* | Sapotaceae | SB | 0.655 | 10 | 0 | 0 | 10 |
| *Chytranthus sp.* | Sapindaceae | SB | unknown | 1 | 0 | 0 | 1 |
| *Cleistopholis glauca* | Annonaceae | P | 0.309 | 13 | 10 | 3 | 26 |
| *Cleistopholis patens* | Annonaceae | P | 0.335 | 3 | 0 | 0 | 3 |
| *Coelocaryon preussii* | Myristicaceae | NPLD | 0.495 | 19 | 4 | 11 | 34 |
| *Coffea arabica* | Rubiaceae | SB | 0.620 | 1 | 0 | 0 | 1 |
| *Cola acuminata* | Malvaceae | SB | 0.500 | 0 | 5 | 0 | 5 |
| *Cola altissima* | Malvaceae | SB | 0.601 | 13 | 0 | 3 | 16 |
| *Cola balayi* | Malvaceae | SB | 0.58 | 0 | 1 | 0 | 1 |
| *Cola cf gigantea* | Malvaceae | SB | 0.601 | 1 | 0 | 0 | 1 |
| *Cola cordifolia* | Sterculiaceae | unknown | 0.523 | 0 | 0 | 9 | 9 |
| *Cola lateritia* | Malvaceae | SB | 0.507 | 4 | 3 | 0 | 7 |
| *Cola nitida* | Malvaceae | SB | 0.601 | 4 | 0 | 5 | 9 |
| *Copaifera mildbraedii* | Fabaceae | NPLD | 0.660 | 1 | 1 | 0 | 2 |
| *Cordia platythyrsa* | Boraginaceae | SB | 0.517 | 0 | 3 | 0 | 3 |
| *Cordia sp.* | Boraginaceae | P | 0.437 | 0 | 1 | 0 | 1 |
| *Corynanthe pachyceras* | Rubiaceae | SB | 0.672 | 28 | 17 | 8 | 53 |
| *Coula edulis* | Olacaceae | SB | 0.895 | 0 | 0 | 46 | 46 |
| *Croton macrostachyus* | Euphorbiaceae | P | 0.479 | 0 | 4 | 0 | 4 |
| *Crudia gabonensis* | Fabaceae | SB | 0.783 | 0 | 2 | 0 | 2 |
| *Cylicodiscus gabunensis* | Fabaceae | P | 0.779 | 10 | 6 | 5 | 21 |
| *Cyrtogonone argentea* | Euphorbiaceae | unknown | unknown | 0 | 0 | 7 | 7 |
| *Dacryodes edulis* | Burseraceae | NPLD | 0.516 | 5 | 4 | 1 | 10 |
| *Dacryodes igaganga* | Burseraceae | NPLD | 0.537 | 0 | 0 | 4 | 4 |
| *Dacryodes macrophylla* | Burseraceae | SB | 0.553 | 0 | 0 | 14 | 14 |
| *Desbordesia glaucescens* | Irvingiaceae | SB | 0.915 | 81 | 54 | 31 | 166 |
| *Desplatsia dewevrei* | Tiliaceae | NPLD | unknown | 0 | 0 | 1 | 1 |
| *Desplatsia sp.* | Malvaceae | NPLD | unknown | 9 | 7 | 0 | 16 |
| *Detarium macrocarpum* | Fabaceae | P | 0.710 | 0 | 1 | 1 | 2 |
| *Dialium bipindense* | Fabaceae | SB | 0.959 | 0 | 0 | 22 | 22 |
| *Dialium pachyphyllum* | Fabaceae | SB | 0.922 | 23 | 0 | 4 | 27 |
| *Dialium sp.* | Fabaceae | SB | 0.823 | 5 | 0 | 0 | 5 |
| *Dialium zenkeri* | Fabaceae | SB | 0.823 | 0 | 2 | 0 | 2 |
| *Dichostemma glaucescens* | Euphorbiaceae | P | unknown | 0 | 0 | 45 | 45 |
| *Diogoa zenkeri* | Olacaceae | SB | 0.696 | 0 | 0 | 13 | 13 |
| *Diospyros canaliculata* | Ebenaceae | SB | 0.698 | 53 | 0 | 0 | 53 |
| *Diospyros crassiflora* | Ebenaceae | SB | 0.858 | 14 | 2 | 1 | 17 |
| *Diospyros mannii* | Ebenaceae | SB | 0.813 | 1 | 0 | 0 | 1 |
| *Diospyros simulans* | Ebenaceae | SB | unknown | 0 | 2 | 10 | 12 |
| *Diospyros sp.* | Ebenaceae | SB | 0.697 | 4 | 1 | 9 | 14 |
| *Discoglypremna caloneura* | Euphorbiaceae | P | 0.340 | 11 | 14 | 3 | 28 |
| *Distemonanthus benthamianus* | Fabaceae | P | 0.605 | 0 | 0 | 13 | 13 |
| *Dracaena arborea* | Dracaenaceae | P | 0.418 | 3 | 0 | 0 | 3 |
| *Drypetes gossweileri* | Putranjivaceae | SB | 0.669 | 12 | 7 | 0 | 19 |
| *Drypetes leonensis* | Putranjivaceae | SB | 0.707 | 0 | 2 | 0 | 2 |
| *Drypetes preussii* | Putranjivaceae | SB | 0.707 | 0 | 4 | 0 | 4 |
| *Drypetes sp.* | Putranjivaceae | SB | 0.707 | 57 | 3 | 0 | 60 |
| *Drypetes spinosa* | Euphorbiaceae | SB | 0.707 | 17 | 0 | 0 | 17 |
| *Duboscia macrocarpa* | Malvaceae | NPLD | unknown | 36 | 20 | 23 | 79 |
| *Duguetia confinis* | Annonaceae | NPLD | 0.621 | 0 | 2 | 0 | 2 |
| *Duguetia staudtii* | Annonaceae | NPLD | 0.643 | 15 | 0 | 5 | 20 |
| *Endodesmia calophylloides* | Clusiaceae | unknown | 0.679 | 0 | 0 | 3 | 3 |
| *Entandrophragma candollei* | Meliaceae | NPLD | 0.574 | 3 | 0 | 0 | 3 |
| *Entandrophragma cf congoense* | Meliaceae | unknown | 0.536 | 1 | 0 | 0 | 1 |
| *Entandrophragma cylindricum* | Meliaceae | NPLD | 0.572 | 21 | 25 | 0 | 46 |
| *Entandrophragma utile* | Meliaceae | NPLD | 0.537 | 1 | 2 | 1 | 4 |
| *Eribroma oblongum* | Malvaceae | SB | 0.638 | 11 | 7 | 9 | 27 |
| *Eriocoelum macrocarpum* | Sapindaceae | SB | 0.523 | 0 | 5 | 0 | 5 |
| *Erismadelphus exsul* | Vochysiaceae | unknown | 0.607 | 7 | 1 | 0 | 8 |
| *Erythrina sp.* | Fabaceae | P | 0.288 | 1 | 0 | 0 | 1 |
| *Erythrophleum ivorense* | Fabaceae | P | 0.774 | 0 | 0 | 6 | 6 |
| *Erythrophleum suaveolens* | Fabaceae | P | 0.824 | 23 | 20 | 0 | 43 |
| *Fernandoa adolfi-friderici* | Bignoniaceae | P | 0.505 | 4 | 2 | 0 | 6 |
| *Fernandoa ferdinandi* | Bignoniaceae | unknown | 0.505 | 0 | 1 | 0 | 1 |
| *Fillaeopsis discophora* | Fabaceae | unknown | 0.501 | 0 | 1 | 3 | 4 |
| *Funtumia africana* | Apocynaceae | SB | 0.424 | 2 | 0 | 0 | 2 |
| *Funtumia elastica* | Apocynaceae | SB | 0.424 | 42 | 38 | 12 | 92 |
| *Garcinia mannii* | Clusiaceae | SB | 0.824 | 0 | 1 | 0 | 1 |
| *Garcinia staudtii* | Clusiaceae | SB | 0.757 | 0 | 3 | 0 | 3 |
| *Gilbertiodendron dewevrei* | Fabaceae | SB | 0.707 | 0 | 0 | 1 | 1 |
| *Greenwayodendron suaveolens* | Annonaceae | SB | 0.695 | 312 | 141 | 15 | 468 |
| *Grewia coriacea* | Tiliaceae | SB | 0.437 | 0 | 0 | 54 | 54 |
| *Grewia spp.* | Malvaceae | SB | 0.557 | 2 | 0 | 0 | 2 |
| *Guarea cedrata* | Meliaceae | NPLD | 0.510 | 3 | 1 | 5 | 9 |
| *Guarea thompsonii* | Meliaceae | NPLD | 0.552 | 3 | 1 | 1 | 5 |
| *Hallea sp.* | Rubiaceae | NPLD | 0.472 | 16 | 0 | 0 | 16 |
| *Hannoa klaineana* | Simaroubaceae | unknown | unknown | 0 | 1 | 0 | 1 |
| *Heisteria parvifolia* | Erythropalaceae | SB | 0.696 | 16 | 0 | 0 | 16 |
| *Hensia trinata* | unknown | SB | unknown | 0 | 1 | 0 | 1 |
| *Hexalobus crispiflorus* | Annonaceae | SB | 0.484 | 14 | 26 | 5 | 45 |
| *Holoptelea grandis* | Ulmaceae | unknown | 0.594 | 0 | 2 | 0 | 2 |
| *Homalium letestui* | Flacourtiaceae | NPLD | 0.714 | 0 | 0 | 15 | 15 |
| *Homalium sp.* | Salicaceae | NPLD | 0.708 | 7 | 31 | 0 | 38 |
| *Hylodendron gabunense* | Fabaceae | NPLD | 0.787 | 71 | 39 | 0 | 110 |
| *Hymenocardia chevalieri* | Phyllanthaceae | NPLD | 0.702 | 0 | 0 | 2 | 2 |
| *Hypodaphnis zenkeri* | Lauraceae | SB | unknown | 0 | 0 | 2 | 2 |
| *Irvingia excelsa* | Irvingiaceae | SB | 0.803 | 4 | 0 | 0 | 4 |
| *Irvingia gabonensis* | Irvingiaceae | SB | 0.775 | 36 | 11 | 11 | 58 |
| *Irvingia grandifolia* | Irvingiaceae | NPLD | 0.800 | 13 | 7 | 4 | 24 |
| *Irvingia robur* | Irvingiaceae | SB | 0.803 | 11 | 5 | 6 | 22 |
| *Irvingia smithii* | Irvingiaceae | SB | 0.803 | 0 | 1 | 0 | 1 |
| *Irvingia sp.* | Irvingiaceae | unknown | 0.803 | 1 | 0 | 0 | 1 |
| *Isolona hexaloba* | Annonaceae | SB | unknown | 10 | 3 | 0 | 13 |
| *Julbernardia seretii* | Fabaceae | SB | 0.689 | 15 | 0 | 0 | 15 |
| *Keayodendron bridelioides* | Phyllanthaceae | SB | unknown | 39 | 17 | 0 | 56 |
| *Khaya anthotheca* | Meliaceae | NPLD | 0.491 | 1 | 1 | 0 | 2 |
| *Khaya ivorensis* | Meliaceae | NPLD | 0.442 | 0 | 1 | 3 | 4 |
| *Kigelia africana* | Bignoniaceae | NPLD | 0.564 | 0 | 0 | 3 | 3 |
| *Klainedoxa gabonensis* | Irvingiaceae | P | 0.926 | 35 | 13 | 5 | 53 |
| *Klainedoxa macrophylla* | Irvingiaceae | P | 0.926 | 0 | 1 | 0 | 1 |
| *Laccodiscus pseudostipularis* | Sapindaceae | SB | unknown | 2 | 0 | 0 | 2 |
| *Lannea welwitschii* | Anacardiaceae | P | 0.405 | 7 | 3 | 1 | 11 |
| *Lasiodiscus fasciculiflorus* | Rhamnaceae | SB | 0.784 | 0 | 0 | 1 | 1 |
| *Lasiodiscus marmoratus* | Rhamnaceae | SB | 0.784 | 0 | 15 | 0 | 15 |
| *Lasiodiscus sp.* | Rhamnaceae | SB | 0.784 | 2 | 0 | 0 | 2 |
| *Lecaniodiscus cupanioides* | Sapindaceae | NPLD | unknown | 5 | 0 | 0 | 5 |
| *Lepidobotrys staudtii* | Lepidobotryaceae | SB | unknown | 3 | 14 | 6 | 23 |
| *Leplaea mayombensis* | Meliaceae | unknown | unknown | 0 | 0 | 1 | 1 |
| *Lophira alata* | Ochnaceae | NPLD | 0.897 | 0 | 0 | 40 | 40 |
| *Lovoa trichilioides* | Meliaceae | NPLD | 0.455 | 2 | 2 | 3 | 7 |
| *Macaranga barteri* | Euphorbiaceae | P | 0.381 | 10 | 1 | 6 | 17 |
| *Macaranga monandra* | Euphorbiaceae | P | 0.381 | 26 | 0 | 0 | 26 |
| *Macaranga sp.* | Euphorbiaceae | P | 0.388 | 0 | 43 | 8 | 51 |
| *Macaranga spinosa* | Euphorbiaceae | P | 0.381 | 13 | 0 | 0 | 13 |
| *Maesopsis eminii* | Rhamnaceae | P | 0.384 | 10 | 5 | 0 | 15 |
| *Magnistipula tessmannii* | Chrysobalanaceae | SB | unknown | 0 | 0 | 2 | 2 |
| *Majidea fosteri* | Sapindaceae | unknown | unknown | 0 | 1 | 0 | 1 |
| *Malacantha alnifolia* | Sapotaceae | unknown | unknown | 0 | 4 | 0 | 4 |
| *Mammea africana* | Clusiaceae | SB | 0.627 | 7 | 0 | 1 | 8 |
| *Manilkara sp.* | Sapotaceae | SB | 0.885 | 3 | 0 | 0 | 3 |
| *Mansonia altissima* | Malvaceae | NPLD | 0.564 | 3 | 13 | 0 | 16 |
| *Maprounea membranaceae* | Euphorbiaceae | P | 0.588 | 0 | 2 | 0 | 2 |
| *Maranthes glabra* | Chrysobalanaceae | SB | 0.875 | 0 | 7 | 5 | 12 |
| *Maranthes inermis* | Chrysobalanaceae | SB | 0.817 | 0 | 1 | 0 | 1 |
| *Maranthes sp.* | Chrysobalanaceae | SB | 0.817 | 1 | 1 | 0 | 2 |
| *Mareyopsis longifolia* | Euphorbiaceae | SB | unknown | 0 | 3 | 11 | 14 |
| *Margaritaria discoidea* | Phyllanthaceae | P | 0.720 | 18 | 15 | 2 | 35 |
| *Markhamia lutea* | Bignoniaceae | P | 0.474 | 0 | 11 | 0 | 11 |
| *Markhamia sp.* | Bignoniaceae | P | 0.581 | 2 | 0 | 0 | 2 |
| *Meiocarpidium lepidotum* | Annonaceae | SB | unknown | 0 | 7 | 2 | 9 |
| *Memecylon sp.* | Melastomataceae | SB | 0.813 | 0 | 0 | 1 | 1 |
| *Microdesmis puberula* | Pandaceae | SB | unknown | 0 | 0 | 6 | 6 |
| *Milicia excelsa* | Moraceae | P | 0.574 | 2 | 0 | 2 | 4 |
| *Millettia laurentii* | Fabaceae | unknown | 0.761 | 0 | 0 | 11 | 11 |
| *Millettia sp.* | Fabaceae | unknown | 0.738 | 0 | 0 | 1 | 1 |
| *Monodora tenuifolia* | Annonaceae | SB | 0.490 | 0 | 4 | 2 | 6 |
| *Morinda lucida* | Rubiaceae | P | 0.558 | 1 | 1 | 1 | 3 |
| *Musanga cecropioides* | Urticaceae | P | 0.243 | 44 | 47 | 20 | 111 |
| *Myrianthus arboreus* | Urticaceae | P | 0.450 | 10 | 4 | 4 | 18 |
| *Nauclea diderrichii* | Rubiaceae | P | 0.674 | 3 | 7 | 0 | 10 |
| *Nauclea pobeguinii* | Rubiaceae | P | 0.674 | 0 | 8 | 0 | 8 |
| *Nesogordonia papaverifera* | Malvaceae | SB | 0.645 | 9 | 11 | 1 | 21 |
| *Oddoniodendron normandii* | Fabaceae | SB | 0.918 | 0 | 6 | 0 | 6 |
| *Odyendyea gabonensis* | Simaroubaceae | NPLD | 0.325 | 50 | 0 | 0 | 50 |
| *Omphalocarpum elatum* | Sapotaceae | SB | 0.550 | 2 | 1 | 0 | 3 |
| *Oncoba brevipe* | Salicaceae | P | 0.580 | 0 | 1 | 0 | 1 |
| *Oncoba glauca* | Salicaceae | P | 0.580 | 12 | 2 | 22 | 36 |
| *Ongokea gore* | Olacaceae | SB | 0.749 | 15 | 0 | 3 | 18 |
| *Ouratea sp.* | Ochnaceae | SB | 0.727 | 0 | 1 | 0 | 1 |
| *Pachyelasma tessmannii* | Fabaceae | SB | 0.738 | 1 | 0 | 0 | 1 |
| *Pachypodanthium staudtii* | Annonaceae | NPLD | unknown | 0 | 5 | 0 | 5 |
| *Pancovia laurentii* | Sapindaceae | SB | unknown | 7 | 0 | 0 | 7 |
| *Panda oleosa* | Pandaceae | SB | 0.565 | 71 | 19 | 15 | 105 |
| *Parkia bicolor* | Fabaceae | NPLD | 0.463 | 1 | 2 | 8 | 11 |
| *Pausinystalia macroceras* | Rubiaceae | SB | 0.588 | 101 | 2 | 23 | 126 |
| *Pentaclethra macrophylla* | Fabaceae | NPLD | 0.841 | 80 | 13 | 3 | 96 |
| *Pentadesma butyracea* | Clusiaceae | SB | 0.806 | 13 | 0 | 0 | 13 |
| *Pericopsis elata* | Fabaceae | P | 0.639 | 4 | 3 | 0 | 7 |
| *Petersianthus macrocarpus* | Lecythidaceae | NPLD | 0.677 | 95 | 34 | 14 | 143 |
| *Phyllanthus discoides* | Lecythidiaceae | P | 0.750 | 0 | 0 | 4 | 4 |
| *Phyllocosmus africanus* | Ixonanthaceae | SB | 0.780 | 34 | 6 | 0 | 40 |
| *Picralima nitida* | Apocynaceae | SB | 0.775 | 0 | 10 | 13 | 23 |
| *Piptadeniastrum africanum* | Fabaceae | NPLD | 0.605 | 23 | 4 | 6 | 33 |
| *Plagiostyles africana* | Euphorbiaceae | NPLD | 0.741 | 61 | 0 | 28 | 89 |
| *Pouteria altissima* | Sapotaceae | NPLD | 0.462 | 0 | 2 | 0 | 2 |
| *Pouteria aningeri* | Sapotaceae | NPLD | 0.462 | 0 | 0 | 1 | 1 |
| *Prioria oxyphylla* | Fabaceae | NPLD | 0.570 | 4 | 0 | 0 | 4 |
| *Pseudospondias microcarpa* | Anacardiaceae | P | unknown | 1 | 1 | 2 | 4 |
| *Psydrax arnoldiana* | Rubiaceae | P | 0.663 | 2 | 4 | 0 | 6 |
| *Pteleopsis hylodendron* | Combretaceae | NPLD | 0.678 | 8 | 5 | 2 | 15 |
| *Pterocarpus soyauxii* | Fabaceae | NPLD | 0.658 | 31 | 10 | 6 | 47 |
| *Pterygota sp.* | Malvaceae | unknown | 0.536 | 26 | 3 | 0 | 29 |
| *Pycnanthus angolensis* | Myristicaceae | NPLD | 0.409 | 24 | 6 | 20 | 50 |
| *Pycnanthus marchalianus* | Myristicaceae | NPLD | 0.409 | 2 | 0 | 0 | 2 |
| *Quassia gabonensis* | Simaroubaceae | NPLD | 0.274 | 0 | 0 | 6 | 6 |
| *Rauvolfia macrophylla* | Apocynaceae | P | 0.475 | 0 | 9 | 0 | 9 |
| *Rauvolfia vomitoria* | Apocynaceae | P | 0.475 | 9 | 7 | 2 | 18 |
| *Rhabdophyllum affine* | Ochnaceae | SB | unknown | 1 | 0 | 0 | 1 |
| *Rhodognaphalon brevicuspe* | Malvaceae | P | unknown | 0 | 1 | 0 | 1 |
| *Ricinodendron heudelotii* | Euphorbiaceae | P | 0.211 | 11 | 9 | 0 | 20 |
| *Rinorea oblongifolia* | Violaceae | SB | 0.671 | 90 | 9 | 0 | 99 |
| *Rinorea sp.* | Violaceae | SB | 0.671 | 0 | 0 | 4 | 4 |
| *Rothmannia acuminata* | Rubiaceae | unknown | 0.641 | 9 | 0 | 0 | 9 |
| *Santiria trimera* | Burseraceae | SB | 0.546 | 47 | 14 | 18 | 79 |
| *Sapium ellipticum* | Euphorbiaceae | P | 0.551 | 2 | 0 | 9 | 11 |
| *Sapium sp.* | Euphorbiaceae | P | 0.551 | 0 | 5 | 2 | 7 |
| *Scottellia mimfiensis* | Flacourtiaceae | SB | 0.572 | 0 | 1 | 1 | 2 |
| *Scyphocephalium ochocoa* | Myristicaceae | NPLD | 0.544 | 0 | 1 | 0 | 1 |
| *Sorindeia grandifolia* | Anacardiaceae | SB | unknown | 0 | 0 | 9 | 9 |
| *Sorindeia sp.* | Anacardiaceae | SB | 0.560 | 15 | 13 | 0 | 28 |
| *Spathodea campanulata* | Bignoniaceae | P | 0.351 | 1 | 0 | 0 | 1 |
| *Staudtia kamerunensis* | Myristicaceae | SB | 0.797 | 21 | 1 | 21 | 43 |
| *Sterculia rhinopetala* | Malvaceae | SB | 0.673 | 0 | 23 | 0 | 23 |
| *Sterculia tragacantha* | Malvaceae | P | 0.427 | 12 | 12 | 2 | 26 |
| *Stereospermum acuminatissimum* | Bignoniaceae | unknown | 0.688 | 0 | 1 | 0 | 1 |
| *Streblus usambarensis* | Moraceae | unknown | 0.771 | 0 | 1 | 0 | 1 |
| *Strephonema pseudocola* | Combretaceae | SB | 0.634 | 0 | 0 | 1 | 1 |
| *Strombosia grandifolia* | Erythropalaceae | SB | 0.825 | 7 | 0 | 2 | 9 |
| *Strombosia pustulata* | Erythropalaceae | SB | 0.830 | 103 | 74 | 26 | 203 |
| *Strombosiopsis tetrandra* | Erythropalaceae | SB | 0.663 | 86 | 23 | 67 | 176 |
| *Strychnos sp.* | Loganiaceae | unknown | 0.696 | 2 | 0 | 0 | 2 |
| *Symphonia globulifera* | Clusiaceae | SB | 0.600 | 3 | 0 | 0 | 3 |
| *Synsepalum caloneura* | Sapotaceae | SB | 0.678 | 0 | 2 | 0 | 2 |
| *Synsepalum dulcificum* | Sapotaceae | SB | 0.678 | 0 | 2 | 0 | 2 |
| *Synsepalum sp.* | Sapotaceae | SB | 0.678 | 10 | 0 | 0 | 10 |
| *Syzygium rowlandii* | Myrtaceae | unknown | 0.632 | 0 | 4 | 0 | 4 |
| *Syzygium spp.* | Myrtaceae | unknown | 0.649 | 2 | 0 | 0 | 2 |
| *Tabernaemontana crassa* | Apocynaceae | P | 0.550 | 23 | 11 | 4 | 38 |
| *Terminalia superba* | Combretaceae | P | 0.459 | 34 | 43 | 6 | 83 |
| *Tessmannia africana* | Fabaceae | NPLD | 0.824 | 52 | 0 | 0 | 52 |
| *Tessmannia anomala* | Fabaceae | SB | 0.799 | 0 | 8 | 0 | 8 |
| *Tetraberlinia bifoliolata* | Fabaceae | SB | 0.512 | 0 | 0 | 6 | 6 |
| *Tetrapleura tetraptera* | Fabaceae | P | 0.570 | 6 | 1 | 0 | 7 |
| *Tetrorchidium didymostemon* | Euphorbiaceae | P | 0.439 | 9 | 3 | 0 | 12 |
| *Treculia africana* | Moraceae | NPLD | unknown | 5 | 0 | 0 | 5 |
| *Trichilia dregeana* | Meliaceae | unknown | 0.482 | 0 | 17 | 11 | 28 |
| *Trichilia monadelpha* | Meliaceae | SB | 0.481 | 12 | 1 | 0 | 13 |
| *Trichilia rubescens* | Meliaceae | SB | 0.634 | 46 | 2 | 0 | 48 |
| *Trichilia sp.* | Meliaceae | SB | 0.634 | 7 | 0 | 0 | 7 |
| *Trichilia tessmannii* | Meliaceae | SB | 0.634 | 25 | 3 | 6 | 34 |
| *Trichilia welwitschii* | Meliaceae | SB | 0.634 | 0 | 14 | 0 | 14 |
| *Trichoscypha acuminata* | Anacardiaceae | NPLD | 0.644 | 8 | 8 | 2 | 18 |
| *Trichoscypha arborea* | Anacardiaceae | unknown | 0.644 | 3 | 0 | 0 | 3 |
| *Trichoscypha cf patens* | Anacardiaceae | NPLD | 0.644 | 1 | 0 | 0 | 1 |
| *Trichoscypha oddonii* | Anacardiaceae | NPLD | 0.644 | 11 | 0 | 0 | 11 |
| *Trichoscypha sp.* | Anacardiaceae | unknown | 0.644 | 1 | 0 | 0 | 1 |
| *Triplochiton scleroxylon* | Malvaceae | P | 0.335 | 5 | 24 | 0 | 29 |
| *Turraeanthus africanus* | Meliaceae | SB | 0.495 | 1 | 0 | 0 | 1 |
| *Uapaca sp.* | Phyllanthaceae | P | 0.634 | 80 | 13 | 11 | 104 |
| *Vernonia conferta* | Asteraceae | P | 0.330 | 0 | 0 | 1 | 1 |
| *Vitex grandifolia* | Lamiaceae | P | 0.424 | 0 | 2 | 10 | 12 |
| *Vitex rivularis* | Lamiaceae | P | 0.549 | 0 | 10 | 0 | 10 |
| *Vitex sp.* | Lamiaceae | unknown | 0.549 | 24 | 0 | 0 | 24 |
| *Xylopia aethiopica* | Annonaceae | P | 0.442 | 22 | 6 | 17 | 45 |
| *Xylopia aurantiiodora* | Annonaceae | P | 0.594 | 0 | 2 | 0 | 2 |
| *Xylopia hypolampra* | Annonaceae | P | 0.640 | 20 | 7 | 0 | 27 |
| *Xylopia quintasii* | Annonaceae | SB | 0.763 | 37 | 26 | 39 | 102 |
| *Xylopia rubescens* | Annonaceae | unknown | 0.594 | 0 | 0 | 5 | 5 |
| *Xylopia sp.* | Annonaceae | unknown | 0.594 | 7 | 8 | 0 | 15 |
| *Xylopia staudtii* | Annonaceae | SB | 0.401 | 2 | 4 | 0 | 6 |
| *Xylopia villosa* | Annonaceae | unknown | 0.594 | 2 | 0 | 0 | 2 |
| *Zanthoxylum buesgenii* | Rutaceae | unknown | 0.586 | 0 | 1 | 0 | 1 |
| *Zanthoxylum gilletii* | Rutaceae | P | 0.686 | 0 | 0 | 5 | 5 |
| *Zanthoxylum heitzii* | Rutaceae | P | 0.450 | 4 | 1 | 2 | 7 |
| *Zanthoxylum lemairei* | Rutaceae | P | 0.686 | 0 | 0 | 1 | 1 |
| *Zanthoxylum macrophylla* | Rutaceae | P | 0.610 | 15 | 0 | 0 | 15 |
| *Zanthoxylum tessmannii* | Rutaceae | P | 0.686 | 0 | 0 | 1 | 1 |

Species are listed in alphabetic order. ^a^Regeneration guild. ^b^Wood-specific gravity (g/cm³). P = Pioneer. NPLD = Non-Pioneer Light-Demander. SB = Shade-Bearer.

**Table S3.** Dates BP of 60 charcoal samples, ranked in increasing order of soil depth for each study area.

| \| Lab. Nr^a^ \| Depth (cm) \| D^b^ \| 68.2% \| 95.4% \| \| --- \| --- \| --- \| --- \| --- \| \| Area 1 \|  \|  \|  \|  \| \| Poz-49319 \| 5 \| 124.79 ± 0.39 \| Modern \|  \| \| Poz-49399 \| 5 \| 113.33 ± 0.35 \| Modern \|  \| \| Poz-49314 \| 5 \| 205 ± 35 \| 297/0 \| 309/0 \| \| Poz-49316 \| 15 \| 210 ± 30 \| 299/0 \| 305/0 \| \| Poz-49322 \| 30 \| 1805 ± 35 \| 1812/1703 \| 1824/1620 \| \| Poz-49320 \| 45 \| 1585 ± 35 \| 1520/1392 \| 1526/1372 \| \| Poz-49321 \| 45 \| 1645 ± 30 \| 1527/1415 \| 1551/1401 \| \| Poz-49315 \| 45 \| 1545 ± 30 \| 1598/1524 \| 1618/1416 \| \| Poz-49318 \| 45 \| 1670 ± 35 \| 1610/1538 \| 1695/1423 \| \| Poz-49398 \| 65 \| 2175 ± 35 \| 2303/2125 \| 2313/2064 \| \| Area 2 \|  \|  \|  \|  \| \| Poz-49337 \| 5 \| 1825 ± 35 \| 1813/1720 \| 1865/1630 \| \| Poz-49340 \| 5 \| 2745 ± 30 \| 2862/2792 \| 2924/2768 \| \| Poz-49342 \| 5 \| 2190 ± 30 \| 2306/2148 \| 2311/2128 \| \| Poz-62628 \| 15 \| 2090 ± 30 \| 2113/2006 \| 2145/1992 \| \| Poz-62629 \| 15 \| 175 ± 35 \| Modern \|  \| \| Poz-62626 \| 15 \| 1810 ± 30 \| 1810/1708 \| 1822/1628 \| \| Poz-62627 \| 15 \| 860 ± 30 \| 791/732 \| 901/694 \| \| Poz-62630 \| 15 \| 305 ± 30 \| 429/306 \| 462/300 \| \| Poz-62637 \| 15 \| 140 ± 30 \| Modern \|  \| \| Poz-62639 \| 15 \| 1745 ± 30 \| 1700/1618 \| 1720/1565 \| \| Poz-62640 \| 15 \| 80 ± 30 \| Modern \|  \| \| Poz-62636 \| 15 \| 2195 ± 30 \| 2306/2151 \| 2313/2133 \| \| Poz-49339 \| 25 \| 1915 ± 30 \| 1888/1825 \| 1934/1742 \| \| Poz-62634 \| 25 \| 360 ± 30 \| 484/325 \| 500/315 \| \| Poz-62631 \| 25 \| 1610 ± 35 \| 1552/1417 \| 1568/1406 \| \| Poz-62638 \| 25 \| 260 ± 30 \| 420/156 \| 430/0 \| \| Poz-62635 \| 25 \| 1775 ± 30 \| 1727/1622 \| 1812/1611 \| \| Poz-49338 \| 35 \| 2220 ± 30 \| 2310/2160 \| 2325/2153 \| \| Poz-49341 \| 35 \| 1670 ± 35 \| 1610/1538 \| 1695/1423 \| \| Poz-49343 \| 35 \| 2165 ± 30 \| 2302/2121 \| 2309/2062 \| \| Poz-62632 \| 35 \| 2265 ± 30 \| 2342/2186 \| 2349/2159 \| \| Poz-62625 \| 55 \| 1750 ± 30 \| 1705/1618 \| 1728/1565 \| \| Poz-49344 \| 75 \| 2250 ± 35 \| 2336/2181 \| 2346/2156 \| \| Poz-49325 \| 105 \| 9400 ± 50 \| 10693/10576 \| 10749/10508 \| \| Poz-49345 \| 145 \| 2275 ± 30 \| 2347/2207 \| 2351/2160 \| \| Area 3 \|  \|  \|  \|  \| \| Poz-62651 \| 5 \| 1860 ± 30 \| 1860/1736 \| 1870/1720 \| \| Poz-62657 \| 5 \| 1720 ± 30 \| 1692/1569 \| 1702/1560 \| \| Poz-49323 \| 10 \| 260 ± 30 \| 420/156 \| 430/0 \| \| Poz-62641 \| 15 \| 1805 ± 30 \| 1810/1705 \| 1822/1626 \| \| Poz-62646 \| 15 \| 1870 ± 30 \| 1868/1740 \| 1877/1724 \| \| Poz-62642 \| 15 \| 2250 ± 30 \| 2334/2182 \| 2345/2156 \| \| Poz-62644 \| 15 \| 2230 ± 30 \| 2316/2160 \| 2334/2154 \| \| Poz-62645 \| 15 \| 790 ± 30 \| 728/686 \| 760/672 \| \| Poz-62647 \| 15 \| 1810 ± 30 \| 1810/1708 \| 1822/1628 \| \| Poz-62648 \| 15 \| 1960 ± 30 \| 1945/1877 \| 1990/1830 \| \| Poz-62649 \| 15 \| 1655 ± 30 \| 1598/1530 \| 1688/1420 \| \| Poz-62650 \| 15 \| 1705 ± 30 \| 1690/1560 \| 1698/1549 \| \| Poz-62654 \| 15 \| 1660 ± 30 \| 1602/1532 \| 1690/1421 \| \| Poz-49334 \| 20 \| 150 ± 25 \| Modern \|  \| \| Poz-49330 \| 25 \| 145 ± 30 \| Modern \|  \| \| Poz-62653 \| 25 \| 1865 ± 30 \| 1864/1738 \| 1874/1720 \| \| Poz-62655 \| 25 \| 1495 ± 30 \| 1405/1347 \| 1516/1310 \| \| Poz-62656 \| 25 \| 625 ± 30 \| 652/559 \| 660/551 \| \| Poz-49335 \| 30 \| 1540 ± 30 \| 1519/1386 \| 1524/1362 \| \| Poz-49324 \| 30 \| 1810 ± 30 \| 1810/1708 \| 1822/1628 \| \| Poz-49331 \| 35 \| 1705 ± 35 \| 1690/1559 \| 1701/1546 \| \| Poz-49333 \| 35 \| 1790 ± 30 \| 1805/1628 \| 1817/1620 \| \| Poz-49327 \| 70 \| 2160 ± 30 \| 2302/2117 \| 2308/2058 \| \| Poz-49328 \| 90 \| 2005 ± 30 \| 1992/1926 \| 2038/1882 \| \| Poz-49329 \| 150 \| 2090 ± 35 \| 2114/2005 \| 2152/1952 \|   ^a^Sample code. ^b^Date BP. The two last columns show confidence intervals (68.2% and 95.4%) for calibrated dates BP. Modern: calibration was not possible because sample was too recent. |  |  |  |  |  |
| --- | --- | --- | --- | --- | --- | --- | --- | --- | --- | --- | --- | --- | --- | --- | --- | --- | --- | --- | --- | --- | --- | --- | --- | --- | --- | --- | --- | --- | --- | --- | --- | --- | --- | --- | --- | --- | --- | --- | --- | --- | --- | --- | --- | --- | --- | --- | --- | --- | --- | --- | --- | --- | --- | --- | --- | --- | --- | --- | --- | --- | --- | --- | --- | --- | --- | --- | --- | --- | --- | --- | --- | --- | --- | --- | --- | --- | --- | --- | --- | --- | --- | --- | --- | --- | --- | --- | --- | --- | --- | --- | --- | --- | --- | --- | --- | --- | --- | --- | --- | --- | --- | --- | --- | --- | --- | --- | --- | --- | --- | --- | --- | --- | --- | --- | --- | --- | --- | --- | --- | --- | --- | --- | --- | --- | --- | --- | --- | --- | --- | --- | --- | --- | --- | --- | --- | --- | --- | --- | --- | --- | --- | --- | --- | --- | --- | --- | --- | --- | --- | --- | --- | --- | --- | --- | --- | --- | --- | --- | --- | --- | --- | --- | --- | --- | --- | --- | --- | --- | --- | --- | --- | --- | --- | --- | --- | --- | --- | --- | --- | --- | --- | --- | --- | --- | --- | --- | --- | --- | --- | --- | --- | --- | --- | --- | --- | --- | --- | --- | --- | --- | --- | --- | --- | --- | --- | --- | --- | --- | --- | --- | --- | --- | --- | --- | --- | --- | --- | --- | --- | --- | --- | --- | --- | --- | --- | --- | --- | --- | --- | --- | --- | --- | --- | --- | --- | --- | --- | --- | --- | --- | --- | --- | --- | --- | --- | --- | --- | --- | --- | --- | --- | --- | --- | --- | --- | --- | --- | --- | --- | --- | --- | --- | --- | --- | --- | --- | --- | --- | --- | --- | --- | --- | --- | --- | --- | --- | --- | --- | --- | --- | --- | --- | --- | --- | --- | --- | --- | --- | --- | --- | --- | --- | --- | --- | --- | --- | --- | --- | --- | --- | --- | --- | --- | --- | --- | --- | --- | --- | --- | --- | --- | --- | --- | --- | --- | --- | --- | --- | --- | --- | --- | --- | --- | --- | --- |
|  |  |  |  |  |  |
|  |  |  |  |  |  |

**Table S4a**. Mean site values (computed at the plot level) of each variable tested for its difference between sites (Kruskal-Wallis test) in Area 1.

|  | Site 1 | Site 2 | Site 3 | Site 4 | Site 5 | Site 6 | *P*-value^c^ |
| --- | --- | --- | --- | --- | --- | --- | --- |
| CAI^a^ |  |  |  |  |  |  |  |
| 0-20 cm | 0.29 | 0.29 | 0.16 | 0.18 | 0.11 | 0.14 | *** |
| 20-100 cm | 0.37 | 0.44 | 0.27 | . | 0.30 | 0.16 | *** |
| Functional traits |  |  |  |  |  |  |  |
| WSG^b^ | 0.62 | 0.64 | 0.66 | 0.62 | 0.65 | 0.62 | *** |
| P relative abundance | 0.29 | 0.18 | 0.15 | 0.25 | 0.16 | 0.24 | 0.01** |
| NPLD relative abundance | 0.28 | 0.32 | 0.20 | 0.27 | 0.16 | 0.14 | *** |
| SB relative abundance | 0.43 | 0.50 | 0.65 | 0.47 | 0.68 | 0.62 | *** |
| P relative basal area | 0.34 | 0.24 | 0.19 | 0.31 | 0.28 | 0.41 | 0.01** |
| NPLD relative basal area | 0.36 | 0.43 | 0.35 | 0.37 | 0.21 | 0.20 | *** |
| SB relative basal area | 0.31 | 0.35 | 0.45 | 0.31 | 0.51 | 0.41 | 0.01** |
| Species abundances |  |  |  |  |  |  |  |
| *Afrostyrax lepidophyllus* | 0.25 | 0.65 | 1.00 | 0.35 | 1.70 | 0.45 | 0.09 |
| *Albizia adianthifolia* | 0.95 | 0.10 | 0.05 | 0.80 | 0.20 | 0.05 | *** |
| *Alstonia boonei* | 0.30 | 0.50 | 0.25 | 0.20 | 0.45 | 0.35 | 0.82 |
| *Angylocalyx pynaertii* | 0.00 | 0.15 | 0.35 | 0.85 | 1.80 | 0.75 | *** |
| *Annickia affinis* | 0.80 | 0.15 | 0.65 | 0.25 | 1.00 | 0.40 | 0.03* |
| *Anonidium mannii* | 0.55 | 0.00 | 0.20 | 0.25 | 0.15 | 0.15 | 0.15 |
| *Beilschmiedia sp.* | 0.10 | 0.20 | 0.20 | 0.15 | 0.15 | 0.20 | 0.95 |
| *Celtis adolfi-friderici* | 0.00 | 0.00 | 0.25 | 0.25 | 0.80 | 0.25 | *** |
| *Celtis mildbraedii* | 0.45 | 0.25 | 0.05 | 0.10 | 0.15 | 0.10 | 0.06 |
| *Centroplacus glaucinus* | 0.20 | 0.15 | 0.15 | 0.25 | 0.20 | 0.20 | 1.00 |
| *Corynanthe pachyceras* | 0.10 | 0.00 | 0.20 | 0.20 | 0.50 | 0.40 | 0.05* |
| *Desbordesia glaucescens* | 0.55 | 0.80 | 1.00 | 0.15 | 1.10 | 0.45 | 0.02* |
| *Dialium pachyphyllum* | 0.25 | 0.65 | 0.20 | 0.00 | 0.05 | 0.00 | 0.08 |
| *Diospyros canaliculata* | 0.00 | 0.00 | 0.00 | 0.00 | 2.65 | 0.00 | *** |
| *Drypetes sp.* | 0.20 | 0.15 | 0.45 | 0.70 | 0.50 | 0.85 | 0.11 |
| *Duboscia macrocarpa* | 0.30 | 0.15 | 0.40 | 0.25 | 0.50 | 0.20 | 0.61 |
| *Entandrophragma cylindricum* | 0.05 | 0.25 | 0.00 | 0.25 | 0.05 | 0.45 | 0.07 |
| *Erythrophleum suaveolens* | 0.15 | 0.00 | 0.15 | 0.10 | 0.50 | 0.25 | 0.19 |
| *Funtumia elastica* | 0.05 | 0.30 | 0.10 | 0.60 | 0.65 | 0.40 | 0.01** |
| *Greenwayodendron suaveolens* | 2.85 | 1.10 | 2.75 | 3.10 | 2.85 | 2.95 | 0.06 |
| *Hylodendron gabunense* | 1.70 | 0.25 | 0.30 | 1.00 | 0.30 | 0.00 | *** |
| *Irvingia gabonensis* | 0.35 | 0.10 | 0.15 | 0.10 | 0.45 | 0.65 | 0.08 |
| *Keayodendron bridelioides* | 0.10 | 0.25 | 0.35 | 0.35 | 0.45 | 0.45 | 0.12 |
| *Klainedoxa gabonensis* | 0.35 | 0.20 | 0.30 | 0.30 | 0.20 | 0.40 | 0.68 |
| *Macaranga monandra* | 0.05 | 0.50 | 0.00 | 0.05 | 0.25 | 0.45 | *** |
| *Musanga cecropioides* | 1.85 | 0.00 | 0.20 | 0.00 | 0.15 | 0.00 | *** |
| *Odyendyea gabonensis* | 0.70 | 1.00 | 0.00 | 0.80 | 0.00 | 0.00 | *** |
| *Panda oleosa* | 0.10 | 0.05 | 1.25 | 1.05 | 0.95 | 0.15 | *** |
| *Pausinystalia macroceras* | 0.90 | 0.70 | 0.85 | 1.20 | 0.70 | 0.70 | 0.45 |
| *Pentaclethra macrophylla* | 1.50 | 0.50 | 1.05 | 0.65 | 0.20 | 0.10 | *** |
| *Petersianthus macrocarpus* | 1.10 | 0.55 | 0.85 | 1.85 | 0.35 | 0.05 | *** |
| *Phyllocosmus africanus* | 0.05 | 0.15 | 0.20 | 0.05 | 1.00 | 0.25 | *** |
| *Piptadeniastrum africanum* | 0.35 | 0.25 | 0.10 | 0.35 | 0.10 | 0.00 | 0.06 |
| *Plagiostyles africana* | 2.45 | 0.60 | 0.00 | 0.00 | 0.00 | 0.00 | *** |
| *Pterocarpus soyauxii* | 0.35 | 0.55 | 0.35 | 0.15 | 0.15 | 0.00 | 0.01** |
| *Pterygota sp.* | 0.05 | 0.00 | 0.10 | 0.35 | 0.50 | 0.30 | *** |
| *Pycnanthus angolensis* | 0.25 | 0.15 | 0.10 | 0.60 | 0.05 | 0.05 | *** |
| *Rinorea oblongifolia* | 0.25 | 2.45 | 1.55 | 0.00 | 0.20 | 0.05 | *** |
| *Santiria trimera* | 1.05 | 0.15 | 0.35 | 0.35 | 0.35 | 0.10 | 0.01** |
| *Staudtia kamerunensis* | 0.10 | 0.25 | 0.10 | 0.40 | 0.15 | 0.05 | 0.08 |
| *Strombosia pustulata* | 0.55 | 0.45 | 0.80 | 0.75 | 1.05 | 1.55 | 0.03* |
| *Strombosiopsis tetrandra* | 1.75 | 0.85 | 0.80 | 0.20 | 0.50 | 0.20 | *** |
| *Tabernaemontana crassa* | 0.05 | 0.15 | 0.15 | 0.15 | 0.15 | 0.50 | 0.05* |
| *Terminalia superba* | 0.00 | 0.05 | 0.00 | 0.25 | 0.45 | 0.95 | *** |
| *Tessmannia africana* | 0.20 | 1.15 | 0.45 | 0.40 | 0.30 | 0.10 | *** |
| *Trichilia rubescens* | 0.55 | 0.00 | 0.65 | 0.30 | 0.55 | 0.25 | 0.03* |
| *Trichilia tessmannii* | 0.10 | 0.00 | 0.00 | 0.45 | 0.30 | 0.40 | *** |
| *Uapaca sp.* | 2.30 | 0.85 | 0.60 | 0.10 | 0.15 | 0.00 | *** |
| *Vitex sp.* | 0.05 | 0.05 | 0.10 | 0.60 | 0.20 | 0.20 | 0.05* |
| *Xylopia aethiopica* | 0.20 | 0.10 | 0.15 | 0.15 | 0.25 | 0.25 | 0.80 |
| *Xylopia hypolampra* | 0.05 | 0.25 | 0.20 | 0.00 | 0.15 | 0.35 | 0.04* |
| *Xylopia quintasii* | 0.05 | 0.10 | 0.40 | 0.20 | 0.75 | 0.35 | *** |
| Family abundances |  |  |  |  |  |  |  |
| Anacardiaceae | 0.30 | 0.35 | 0.45 | 0.70 | 0.35 | 0.40 | 0.38 |
| Annonaceae | 4.75 | 2.05 | 4.65 | 4.60 | 5.95 | 5.40 | *** |
| Apocynaceae | 0.45 | 1.00 | 0.55 | 1.05 | 1.30 | 1.50 | 0.03* |
| Burseraceae | 1.25 | 0.25 | 0.35 | 0.35 | 0.35 | 0.15 | 0.01** |
| Cannabaceae | 0.75 | 0.65 | 0.65 | 0.95 | 1.20 | 0.35 | 0.09 |
| Centroplacaceae | 0.20 | 0.15 | 0.15 | 0.25 | 0.20 | 0.20 | 1.00 |
| Clusiaceae | 0.25 | 0.25 | 0.40 | 0.45 | 0.20 | 0.00 | 0.17 |
| Combretaceae | 0.00 | 0.05 | 0.10 | 0.40 | 0.50 | 1.05 | *** |
| Ebenaceae | 0.15 | 0.00 | 0.35 | 0.10 | 2.75 | 0.25 | *** |
| Erythropalaceae | 2.70 | 1.55 | 1.85 | 1.00 | 1.65 | 1.85 | 0.10 |
| Euphorbiaceae | 2.80 | 1.50 | 0.35 | 0.60 | 1.05 | 1.70 | *** |
| Fabaceae | 5.85 | 4.20 | 3.60 | 5.20 | 4.30 | 2.40 | *** |
| Huaceae | 0.25 | 0.65 | 1.00 | 0.35 | 1.70 | 0.45 | 0.09 |
| Irvingiaceae | 1.60 | 1.20 | 1.85 | 0.65 | 2.05 | 1.70 | 0.05* |
| Ixonanthaceae | 0.05 | 0.15 | 0.20 | 0.05 | 1.00 | 0.25 | *** |
| Lamiaceae | 0.05 | 0.05 | 0.10 | 0.60 | 0.20 | 0.20 | 0.05* |
| Lauraceae | 0.10 | 0.20 | 0.20 | 0.15 | 0.15 | 0.20 | 0.95 |
| Lecythidaceae | 1.10 | 0.55 | 0.85 | 1.85 | 0.35 | 0.05 | *** |
| Malvaceae | 0.80 | 0.35 | 0.80 | 1.30 | 2.00 | 1.60 | *** |
| Meliaceae | 1.85 | 0.70 | 1.15 | 1.40 | 1.00 | 1.15 | 0.06 |
| Myristicaceae | 0.60 | 0.50 | 0.45 | 1.25 | 0.20 | 0.30 | 0.01** |
| Pandaceae | 0.10 | 0.05 | 1.25 | 1.05 | 0.95 | 0.15 | *** |
| Phyllanthaceae | 2.40 | 1.10 | 1.30 | 0.60 | 1.20 | 0.55 | 0.03* |
| Putranjivaceae | 0.30 | 0.40 | 0.55 | 0.70 | 0.50 | 1.00 | 0.36 |
| Rubiaceae | 1.05 | 0.80 | 1.30 | 1.80 | 1.45 | 2.00 | 0.16 |
| Sapindaceae | 0.10 | 0.25 | 0.15 | 0.40 | 0.15 | 0.00 | 0.15 |
| Sapotaceae | 0.30 | 0.30 | 0.40 | 0.60 | 0.05 | 0.00 | 0.01** |
| Simaroubaceae | 0.70 | 1.00 | 0.00 | 0.80 | 0.00 | 0.00 | *** |
| Urticaceae | 2.20 | 0.00 | 0.20 | 0.10 | 0.20 | 0.00 | *** |
| Violaceae | 0.25 | 2.45 | 1.55 | 0.00 | 0.20 | 0.05 | *** |

^a^Charcoal abundance index. ^b^Wood-specific gravity (g/cm³). ^c^*P*-value of a Kruskal-Wallis test among sites: **p-*value < 0.05 ** *p-*value < 0.01 *** *p-*value < 0.001. P/NPLD/SB = Pioneers / Non-Pioneer Light-Demanders / Shade-Bearers. Species and families are listed in alphabetic order.

**Table S4b**. Mean site values (computed at the plot level) of each variable tested for its difference between sites (Kruskal-Wallis test) in Area 2.

|  | Site 7 | Site 8 | Site 9 | *P*-value^c^ |
| --- | --- | --- | --- | --- |
| CAI^a^ |  |  |  |  |
| 0-20 cm | 0.47 | 0.35 | 0.56 | 0.43 |
| 20-100 cm | 0.35 | 0.28 | 0.41 | 0.39 |
| Functional traits |  |  |  |  |
| WSG^b^ | 0.59 | 0.58 | 0.64 | *** |
| P relative abundance | 0.32 | 0.35 | 0.24 | 0.05* |
| NPLD relative abundance | 0.20 | 0.22 | 0.15 | 0.16 |
| SB relative abundance | 0.48 | 0.44 | 0.61 | *** |
| P relative basal area | 0.47 | 0.43 | 0.37 | 0.31 |
| NPLD relative basal area | 0.23 | 0.26 | 0.22 | 0.77 |
| SB relative basal area | 0.30 | 0.31 | 0.41 | 0.03* |
| Species abundances |  |  |  |  |
| *Alstonia boonei* | 2.26 | 0.22 | 0.50 | *** |
| *Annickia affinis* | 0.74 | 1.11 | 1.00 | 0.92 |
| *Anonidium mannii* | 1.32 | 0.39 | 0.31 | 0.01** |
| *Celtis adolfi.friderici* | 1.11 | 0.17 | 0.13 | *** |
| *Desbordesia glaucescens* | 1.79 | 0.56 | 0.63 | 0.23 |
| *Duboscia macrocarpa* | 0.53 | 0.33 | 0.25 | 0.83 |
| *Entandrophragma cylindricum* | 0.37 | 0.22 | 0.88 | 0.12 |
| *Erythrophleum suaveolens* | 0.21 | 0.28 | 0.69 | 0.21 |
| *Funtumia elastica* | 1.21 | 0.61 | 0.25 | 0.01** |
| *Greenwayodendron suaveolens* | 1.89 | 1.67 | 4.69 | 0.06 |
| *Hexalobus crispiflorus* | 0.58 | 0.33 | 0.56 | 0.40 |
| *Homalium sp.* | 0.79 | 0.61 | 0.31 | 0.21 |
| *Hylodendron gabunense* | 0.58 | 1.11 | 0.50 | 0.34 |
| *Macaranga sp.* | 0.89 | 0.72 | 0.81 | 0.52 |
| *Musanga cecropioides* | 0.05 | 2.56 | 0.00 | *** |
| *Petersianthus macrocarpus* | 0.74 | 1.06 | 0.06 | *** |
| *Sterculia rhinopetala* | 1.21 | 0.00 | 0.00 | *** |
| *Strombosia pustulata* | 1.00 | 1.11 | 2.19 | 0.03* |
| *Strombosiopsis tetrandra* | 0.63 | 0.28 | 0.38 | 0.31 |
| *Terminalia superba* | 1.74 | 0.11 | 0.50 | *** |
| *Triplochiton scleroxylon* | 0.74 | 0.44 | 0.13 | 0.03* |
| *Xylopia quintasii* | 0.79 | 0.11 | 0.56 | 0.03* |
| Family abundances |  |  |  |  |
| Anacardiaceae | 0.37 | 0.44 | 0.63 | 0.36 |
| Annonaceae | 6.47 | 4.67 | 8.19 | 0.14 |
| Apocynaceae | 4.37 | 1.72 | 1.00 | *** |
| Cannabaceae | 1.74 | 0.50 | 0.31 | 0.01** |
| Combretaceae | 1.79 | 0.17 | 0.69 | *** |
| Erythropalaceae | 1.63 | 1.39 | 2.56 | 0.07 |
| Euphorbiaceae | 1.58 | 1.44 | 2.50 | 0.22 |
| Fabaceae | 1.89 | 3.33 | 3.50 | 0.16 |
| Irvingiaceae | 2.32 | 1.28 | 1.56 | 0.79 |
| Lecythidaceae | 0.74 | 1.06 | 0.06 | *** |
| Malvaceae | 4.84 | 1.56 | 0.81 | *** |
| Meliaceae | 1.47 | 0.94 | 1.81 | 0.05* |
| Phyllanthaceae | 0.74 | 0.83 | 1.00 | 0.23 |
| Rhamnaceae | 0.79 | 0.06 | 0.25 | 0.04* |
| Rubiaceae | 1.26 | 0.56 | 0.63 | 0.18 |
| Salicaceae | 0.84 | 0.72 | 0.31 | 0.19 |
| Sapotaceae | 0.79 | 0.72 | 0.19 | 0.03* |
| Urticaceae | 0.16 | 2.61 | 0.06 | *** |

^a^Charcoal abundance index. ^b^Wood-specific gravity (g/cm³). ^c^*P*-value of a Kruskal-Wallis test among sites: **p-*value < 0.05 ** *p-*value < 0.01 *** *p-*value < 0.001. P/NPLD/SB = Pioneers / Non-Pioneer Light-Demanders / Shade-Bearers. Species and families are listed in alphabetic order.

**Table S4c**. Mean site values (computed at the plot level) of each variable tested for its difference between sites (Kruskal-Wallis test) in Area 3.

|  | Site 10 | Site 11 | Site 12 | *P*-value^c^ |
| --- | --- | --- | --- | --- |
| CAI^a^ |  |  |  |  |
| 0-20 cm | 0.40 | 0.42 | 0.38 | 0.78 |
| 20-100 cm | 0.38 | 0.36 | 0.55 | 0.19 |
| Functional traits |  |  |  |  |
| WSG^b^ | 0.62 | 0.65 | 0.64 | 0.39 |
| P relative abundance | 0.19 | 0.23 | 0.22 | 0.53 |
| NPLD relative abundance | 0.27 | 0.15 | 0.20 | 0.01** |
| SB relative abundance | 0.55 | 0.62 | 0.58 | 0.46 |
| P relative basal area | 0.26 | 0.28 | 0.27 | 0.97 |
| NPLD relative basal area | 0.42 | 0.25 | 0.32 | 0.04* |
| SB relative basal area | 0.32 | 0.47 | 0.41 | 0.18 |
| Species abundances |  |  |  |  |
| *Alstonia boonei* | 0.83 | 0.64 | 0.42 | 0.88 |
| *Annickia affinis* | 1.50 | 1.73 | 2.17 | 0.24 |
| *Berlinia congolensis* | 0.08 | 1.09 | 0.67 | 0.01** |
| *Blighia welwitschii* | 1.75 | 2.36 | 2.25 | 0.44 |
| *Calpocalyx dinklagei* | 0.08 | 1.09 | 1.08 | 0.04* |
| *Coula edulis* | 0.42 | 1.45 | 2.08 | 0.01** |
| *Desbordesia glaucescens* | 0.75 | 1.82 | 0.17 | 0.04* |
| *Dialium bipindense* | 0.42 | 0.55 | 0.92 | 0.19 |
| *Dichostemma glaucescens* | 0.67 | 0.64 | 2.50 | 0.04* |
| *Duboscia macrocarpa* | 1.00 | 0.36 | 0.58 | 0.84 |
| *Grewia coriacea* | 2.58 | 0.73 | 1.25 | 0.21 |
| *Lophira alata* | 0.92 | 0.45 | 2.00 | 0.64 |
| *Musanga cecropioides* | 0.00 | 0.36 | 1.33 | 0.02* |
| *Oncoba glauca* | 0.25 | 0.64 | 1.00 | 0.28 |
| *Pausinystalia macroceras* | 0.33 | 0.45 | 1.17 | *** |
| *Plagiostyles africana* | 0.42 | 0.82 | 1.17 | 0.24 |
| *Pycnanthus angolensis* | 1.33 | 0.09 | 0.25 | *** |
| *Staudtia kamerunensis* | 0.83 | 0.45 | 0.50 | 0.48 |
| *Strombosia pustulata* | 1.33 | 0.45 | 0.42 | 0.17 |
| *Strombosiopsis tetrandra* | 2.08 | 2.82 | 0.92 | 0.01** |
| *Xylopia quintasii* | 1.42 | 1.64 | 0.33 | 0.33 |
| Family abundances |  |  |  |  |
| Annonaceae | 4.08 | 5.00 | 4.42 | 0.53 |
| Apocynaceae | 1.83 | 0.91 | 0.67 | 0.33 |
| Burseraceae | 1.17 | 1.27 | 1.17 | 0.87 |
| Ebenaceae | 0.33 | 1.09 | 0.33 | 0.41 |
| Erythropalaceae | 3.42 | 3.45 | 1.33 | 0.01** |
| Euphorbiaceae | 2.17 | 4.09 | 5.33 | 0.13 |
| Fabaceae | 3.83 | 5.64 | 4.33 | 0.15 |
| Irvingiaceae | 1.50 | 2.64 | 0.83 | 0.14 |
| Malvaceae | 1.83 | 1.00 | 0.83 | 0.23 |
| Meliaceae | 1.83 | 0.27 | 1.42 | 0.01** |
| Myristicaceae | 2.42 | 0.55 | 1.42 | 0.04* |
| Ochnaceae | 0.92 | 0.45 | 2.00 | 0.64 |
| Olacaceae | 0.58 | 2.27 | 2.50 | 0.02* |
| Pandaceae | 0.75 | 0.91 | 0.17 | 0.10 |
| Phyllanthaceae | 1.00 | 0.82 | 0.67 | 0.95 |
| Rubiaceae | 0.42 | 1.00 | 1.33 | 0.01** |
| Salicaceae | 0.25 | 0.64 | 1.00 | 0.28 |
| Sapindaceae | 1.75 | 2.36 | 2.25 | 0.44 |
| Tiliaceae | 2.58 | 0.82 | 1.25 | 0.25 |
| Urticaceae | 0.08 | 0.45 | 1.50 | 0.03* |

^a^Charcoal abundance index. ^b^Wood-specific gravity (g/cm³). ^c^P-value of a Kruskal-Wallis test among sites: **p-*value < 0.05 ** *p-*value < 0.01 *** *p-*value < 0.001. P/NPLD/SB = Pioneers / Non-Pioneer Light-Demanders / Shade-Bearers. Species and families are listed in alphabetic order.
