# Supplementary material for: Soil Charcoal to Assess the Impacts of Past Human Disturbances on Tropical Forests
Source: PLoS One. 2014 Nov 12;9(11):e108121. doi: 10.1371/journal.pone.0108121 (PMC4229094; doi:10.1371/journal.pone.0108121)
Supplement: Appendix S1 — Contact details of logging companies. (DOCX) [file pone.0108121.s003.docx]

**Appendix S1**

**Area 1**

Name of the company: **Pallisco**

Contact details:

478 rue des Cocotiers

BP 394 Douala CAMEROUN

**Phone**: +237 33-42-54-16 / 33-43-36-98

+237 99-68-14-21 / 77-70-74-17

**Fax**: +237 33-43-31-53

**E-mail**: info@pallisco-cifm.com

exploitation@pallisco-cifm.com.

**Website**: <http://ang.pallisco-cifm.com>

**Area 2**

# Name of the company: SFID (Société Forestière et Industrielle de la Doumé / Doumé Forestry Company)

Contact details:

Rougier SA. 75, Avenue des Champs Elysées

75008 Paris France

Tel.: +33 1 53 77 25 00

**Website**: http://www.rougier.fr/en

**Area 3**

Name of the company : **WIJMA Cameroun S.A.**

Contact details:

Bonanjo B.P. 1616 DOUALA CAMEROUN

Tél.: +237 33 43 07 11 / +237 33 42 80 22

Port.: +237 99 80 04 97

Fax: +237 33 42 33 69

**Website**: www.wijmadouala.com

**Other precisions**

Our field studies did not involve endangered or protected species.
